# The Pawnee earthquake as a result of the interplay among injection, faults and foreshocks

**DOI:** 10.1038/s41598-017-04992-z

**Published:** 2017-07-10

**Authors:** Xiaowei Chen, Nori Nakata, Colin Pennington, Jackson Haffener, Jefferson C. Chang, Xiaohui He, Zhongwen Zhan, Sidao Ni, Jacob I. Walter

**Affiliations:** 1 ConocoPhillips School of Geology and Geophysics, the University of Oklahoma, Norman, OK USA; 2 Oklahoma Geological Survey, the University of Oklahoma, Norman, OK USA; 3 University of Science and Technology of China, Hefei, China; 4 Department of Geological Science, Caltech, Pasadena USA; 5 State Key Laboratory of Geodesy and Earth’s Dynamics, Institute of Geodesy and Geophysics, Chinese Academy of Science, Wuhan, China

## Abstract

The Pawnee M5.8 earthquake is the largest event in Oklahoma instrument recorded history. It occurred near the edge of active seismic zones, similar to other M5+ earthquakes since 2011. It ruptured a previously unmapped fault and triggered aftershocks along a complex conjugate fault system. With a high-resolution earthquake catalog, we observe propagating foreshocks leading to the mainshock within 0.5 km distance, suggesting existence of precursory aseismic slip. At approximately 100 days before the mainshock, two M ≥ 3.5 earthquakes occurred along a mapped fault that is conjugate to the mainshock fault. At about 40 days before, two earthquakes clusters started, with one M3 earthquake occurred two days before the mainshock. The three M ≥ 3 foreshocks all produced positive Coulomb stress at the mainshock hypocenter. These foreshock activities within the conjugate fault system are near-instantaneously responding to variations in injection rates at 95% confidence. The short time delay between injection and seismicity differs from both the hypothetical expected time scale of diffusion process and the long time delay observed in this region prior to 2016, suggesting a possible role of elastic stress transfer and critical stress state of the fault. Our results suggest that the Pawnee earthquake is a result of interplay among injection, tectonic faults, and foreshocks.

## Introduction

A well-established injection experiment in the 1960s demonstrates that human can influence earthquake occurrence by pumping water in the subsurface^1, 2^. Decades later, the central United States has seemingly replicated the 1960s experiment by accident: rapid increase in seismicity rates since 2009 that can not be explained by steady tectonic stress loading^3, 4^. The mechanism is generally explained by the classic Coulomb stress view, where increased pore pressure reduces effective normal stress, and brings faults closer to failure. Induced earthquakes often occur in the form of swarm-like clustering with no clear mainshocks, and often exhibit spatio-temporal migrations that are regarded as the manifestation of fluid propagation^5^. Most previous studies focused on single injection well or wells that are directly linked to a localized sequence, where clear spatio-temporal correlation can be established^6, 7^. However, recent studies suggest that induced earthquakes can occur as far as 20 km away from the injection zone with long-term fluid diffusion^8, 9^. The larger spatial scale of pressure propagation makes it more challenging to understand the mechanism driving earthquake occurrence patterns, especially for large damaging earthquakes, as the expected pressure increase would be much smaller due to the spatial decay^8, 10^.

While it could be explained by critically stressed fault being re-activated by small amplitude of stress perturbation (e.g., kPa), the roles of earthquake-to-earthquake interaction and aseismic slip are often omitted. Statistical modeling to an induced sequence in Arkansas and a natural swarm nearby found that both background seismicity rate and aftershock productivity needed to be increased to match observations for the induced sequence^4^. The increase in background seismicity is naturally expected due to increased external forcing (e.g., pore pressure or aseismic slip)^11^, while the increase in aftershock productivity suggests that inter-event triggering can not be ignored. In Oklahoma, the Mw5.7 Prague earthquake may have been triggered by increased Coulomb stress from the M5.0 foreshock one day before^12^, suggesting that smaller earthquakes can trigger larger events.

In addition to the direct Coulomb stress triggering from a large foreshock, increasing number of aseismic slip triggering mainshocks have been reported for plate boundary earthquakes^13, 14^; however, it is rarely reported for induced seismicity until recently. Aseismic slip within highly pressurized zone was observed during a controlled fluid injection experiment on a natural fault^15^. Microearthquakes were triggered when the aseismic slip exceeded the pressurized zone; therefore, the seismic slip was only an indirect effect of fluid injection. Analysis of an earthquake swarm with two M5 earthquakes in southern California suggested that the M5 earthquakes were likely triggered by an injection-induced aseismic slip^16^. The aseismic slip started on a shallow normal fault following a rapid increase in injection rate two years before the M5 events occurred. Both of these observations suggest that aseismic slip initiates within highly pressurized zones close to injection zones, and triggers subsequent seismic slip.

Some aseismic episodes are observed through direct measurement of ground deformation^16^, but such direct measurement is often unavailable. Most of the studies infer aseismic slip through analysis of foreshock sequences^14, 17^: the “swarm-like” foreshock sequences migrating within the mainshock nucleation zone is often regarded as a signature of aseismic slip. With improved detection and location techniques, increasing number of studies report foreshock migrations reflecting mainshock preparation processes^18–21^. However, as most potentially induced sequences occur as earthquake “swarms”, the role of foreshocks are often not discussed, and all earthquakes are assumed to be triggered by pore pressure.

In this study, we seek to better understand the nucleation processes of large earthquakes in Oklahoma, with the focus on the triggering process of the Pawnee earthquake. We begin by an overview of occurrence patterns of earthquakes in Oklahoma, and their relationship with injection zones. Then, we focus on the Pawnee County with a detailed analysis of the relationship between injection and precursory activities, as well as the stress interactions between the M3+ foreshocks and the mainshock through Coulomb stress analysis.

## Oklahoma earthquakes: tectonics, injection and faults

**Figure 1:**
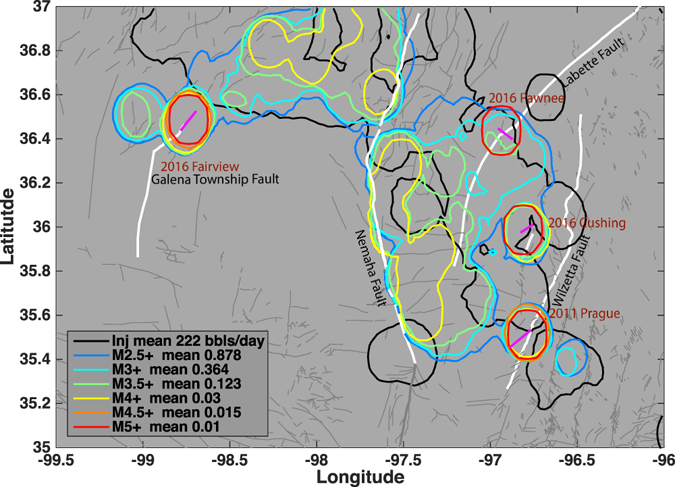
Map view of event density distribution for different sized earthquakes and injection rates. Colored lines show the contour lines of average number of events for each magnitude range (see Legend). Gray lines show mapped faults from OGS database^86^. The major faults are labeled and shown in white thick lines. Thick purple lines denote the seismogenic faults for the four M5+ earthquakes delineated from earthquake locations. Each contour line represents spatial grids that have number of events larger than the average number of events per smoothed bin within the magnitude threshold. Individual density map is shown in Figure S1. The three M5+ sequences are well located at the edge of the seismic zones. The black contour line shows the spatial grids that have average injection rate from 2011 to 2016 higher than the average injection rate per smoothed bin. The figure is generated with Matlab 2015a, available at: http://www.mathworks.com/.

**Figure 2:**
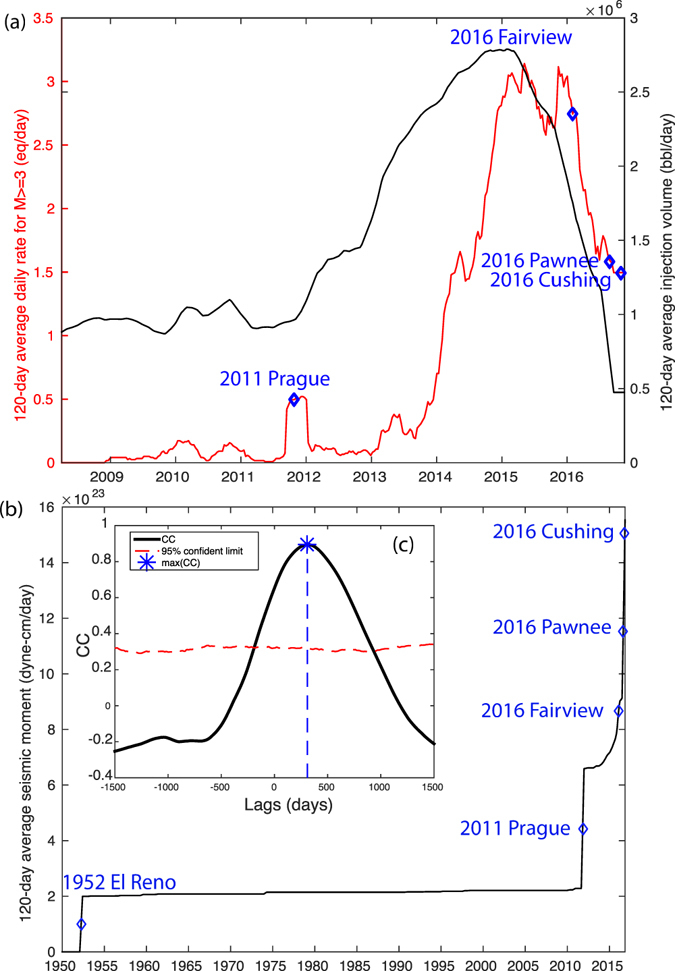
Overview of the relationship between injection and seismicity for Oklahoma. (**a**) Black line shows the history of state-wide daily injection rate (averaged per 120 days) and red line shows the state-wide daily earthquake rate for *M* ≥ 3 earthquakes (averaged per 120 days). Dashed lines show the three M5 sequences since 2010. (**b**) History of cumulative moment release in Oklahoma. The four major sequences are shown with blue diamonds, and corresponding names are labeled. The inset shows the cross-correlation between injection rate and seismicity rate. The blue dashed line shows the lag at maximum correlation. The red line shows the 95% confidence limit of correlation.

Oklahoma is located at the forefront of the mid-continent rift system, which terminates at the Southern Oklahoma Aulacogen^22^, and references therein. The major fault systems in Oklahoma include: the NW-SE trending Meers Fault, the N-S trending Nemaha Fault, the NNE-SSW trending Wilzetta Fault and the NNE-SSW trending Labette Fault (Fig. 1). The basement rocks are likely pervasively fractured due to large scale tectonic activities^23^. Oklahoma has been seismically active compared to neighboring states, with a M7 earthquake along the Meers Fault approximately 1100 years ago; and the overall NE-SW trending seismic zone is consistent with previous observations of seismicity in the 1970s^24, 25^. The total seismic moment release history since the 1950s is dominated by the five M5 earthquake sequences (Fig. 2a): the 1952 M5.5 El Reno earthquake, the 2011 Mw5.7 Prague earthquake with one Mw5.0 foreshock and one Mw5.0 aftershock^26^, the 2016 M5.1 Fairview earthquake preceded by series of M4 earthquakes^9^, the 2016 Mw5.8 Pawnee earthquake with no M4 earthquakes, and the 2016 M5.0 Cushing earthquake occurred on a fault that ruptured in 2015 with two M4 earthquakes. The seismic moment release is relatively low between 1952 and 2010, until the Prague earthquake in 2011, followed by rapid acceleration (Fig. 2).

At present, the consensus is that injection into the Arbuckle Group is the primary cause of induced seismicity^8, 27–29^. The Arbuckle Group is predominately the deepest sediment layer overlying the crystalline basement, and is mostly under-pressured throughout Oklahoma^28^. Therefore, this layer is a favorable formation for wastewater disposal, and most of the injections occur with zero or low pressure. In this study, we confine ourselves to analyze disposal wells that are injecting into the Arbuckle Group.

We first visualize the spatial correlation between injection activities and earthquakes by calculating the contour lines of average density for different sized earthquakes and injection wells (for detailed processing, see Method M1). Key observations from Fig. 1 are summarized below:

- The overall injection and seismicity are divided into two major regions, separated by the Nemaha Fault. Contours of M2.5+ and M3+ earthquakes mostly overlap with injection zones, which supports direct triggering of small earthquakes due to increased pore pressure.
- With increasing magnitude, events are separated into different zones, and localize between injection zones and the Nemaha fault (i.e., east of Nemaha in central Oklahoma, and west of Nemaha in northern Oklahoma). Some isolated patches of M3.5+ and M4+ events exist further away from the Nemaha fault (i.e., close to M5+ contours).
- For M5+ earthquakes since 2011, they are all located at the edge of the injection zones as outlined by the contour line of injection rate based on the Arbuckle Group disposal wells (Fig. 1 and Method M1).
- The large regional faults that are associated with the M5+ earthquakes are strikingly similar to each other in terms of the overall NNE-SSW strike direction. The three earthquakes all occur along the splay or step-over faults in the NE-SW (or ESE-WNW that is conjugate to NE-SW) direction adjacent to the major regional faults.

History of injection in the Arbuckle Group shows two periods of injection (Fig. 2b): (1) 2010 to 2011 with average rate of 1 million barrels/day; (2) between late 2012 and 2016, with peak total rate reaching 2.8 million barrels/day in late 2014. The 30-day smoothing of injection rate and seismicity rate show remarkable similarity. Cross-correlation of the two time-series finds maximum correlation coefficients (CC) of 0.91 with time delay of 300 days. The correlation is significantly above the 95% confident limit, suggesting that this is unlikely to occur by random chance (Fig. 2b inset and Method M3).

The average time delay of 300 days statewide is consistent with the expected time delay from pore pressure diffusion process - the time scale of pressure diffusion is predicted to evolve according to the formulation^30^

1 $$t=\frac{{r}^{2}}{4\pi D},$$

where *r* is the distance from injection point to fault zone, *D* is the diffusivity (*m* ^2^/*s*). With distances of 10 km and diffusivity ranging from 0.05 to 0.5 *m* ^2^/*s* (typical values for seismogenic layers^31^), we get the time delay range from 185 to 1850 days, and the 300 days would be a reasonable value for response to pore pressure changes due to fluid diffusion.

For both periods, decrease in injection rate is followed by decrease in seismicity rate. At present, the injection rate has decreased to the 2012 level, and the seismicity rate has decreased to the 2014 level for M3+ earthquakes (Fig. 2b). The overall decrease in seismicity rate suggests effectiveness in regulation^32^, and demonstrates that injection induced earthquake can be anthropogenically modulated, whether increase or decrease.

However, the four M5 earthquake sequences since 2011 appear to occur during periods with low or decreased injection rate and seismicity rate. The Prague earthquake occurred after the first peak injection and a few months of seismic quiescence in 2011, prior to the ramp-up of injection in 2012. The earthquake rate decreased from 3 in early 2015 to 2.5 events (M ≥3) per day in late 2015, until the Woodward/Fairview clusters started in northwest Oklahoma, which increased the earthquake rate slightly. The seismicity rate gradually decreased again since early 2016, from 3 to 2.6 at the time of the Fairview earthquake. Since the Fairview earthquake in early 2016, seismicity rate has substantially decreased from 2.6 to 1.6 events per day before the Pawnee and later Cushing earthquakes occurred. This means that Fig. 2b can explain the general trend of the statewide seismicity rate variations, but has difficulty accounting for the short-term trend of the larger earthquakes.

Overall, the larger (e.g., M4.5+ and M5+) earthquakes exhibit substantial differences in the spatio-temporal response to injection compared to smaller earthquakes. The delayed occurrence of larger earthquakes after decrease in injection and at further distances from injection zones, have been reported for hydraulic fracturing and enhanced geothermal system^10^. Calibrated geomechanical model using stochastic Coulomb stress triggering found that for models with spatial varying b-value and stress drop that match observations, the probability of larger (*M* ≥ 4) earthquakes is highest after shut-in, and at further distances away from the injection point^10^. The modeling results suggests possible scenarios for Oklahoma earthquakes that can be further tested with future higher-resolution catalogs (lower Mc, higher location accuracy, calibrated magnitudes): at the early stages of injection, highest pore pressure occurs near injection zones, which reduces the differential stress, and can trigger less critically stressed fractures with high b-value^33^; at later stages, the pore pressure distribution becomes more uniform with large scale diffusion, only critically stressed faults can be reactivated with normal b-values close to tectonic values^10, 33^.

**Figure 3:**
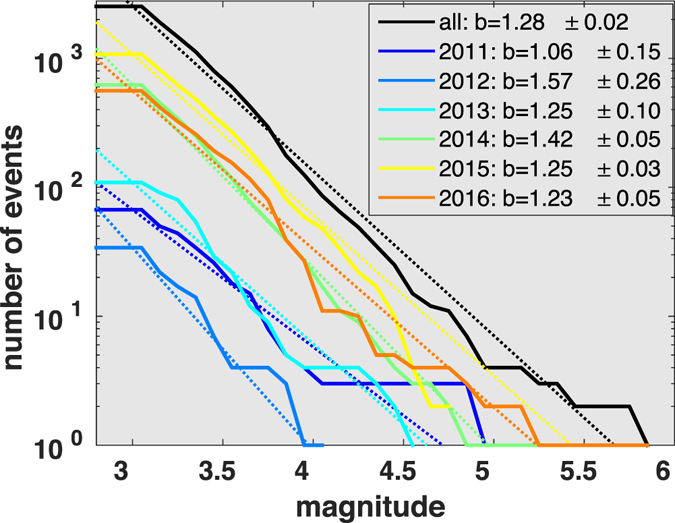
Magnitude frequency distribution for each year since 2011, and all years combined. The b-values and standard deviations are listed in the figure legend.

For both Prague and Fairview sequences, b-values lower than 1 are reported^34, 35^, respectively. For the Pawnee sequence, using only early aftershocks, the b-value is estimated to be 1.06, close to normal tectonic b-values (Figure S2). The normal b-values for the large M5 sequences is consistent with the expected b-value from reactivation of regional tectonic faults. For a given tectonic block, the maximum magnitude can be estimated based on a linear extrapolation of the magnitude-frequency distribution^36^:

2 $${M}_{max}={M}_{c}+\frac{1}{b}{\mathrm{log}}_{10}N,$$

where *b* is the b-value that describes the slope of the power law distribution, *M*_*c*_ is the reference magnitude, and *N* is the number of earthquakes above *M*_*c*_. The statewide catalog is estimated to be complete above 2.5, and we use a reference magnitude of 3 to account for some uncertainties in magnitude estimates, which results in 2000+ M3+ earthquakes since 2011 (Fig. 3). With a b-value of 1.28, the largest expected magnitude is 5.6 (Fig. 3), which is comparable to the magnitude of the Pawnee earthquake.

However, the Pawnee earthquake differs from the two previous M5+ earthquakes in the lack of large magnitude events (i.e., M4+) preceding the mainshock in a relatively short time scale: the M5.7 Prague earthquake was preceded by a M5 earthquake less than one day before^26^; and the M5.1 Fairview earthquake was preceded by 2 M4.5+ earthquakes one month before. The Prague mainshock was likely triggered by the Coulomb stress increase from the M5 foreshock^12^, while the Fairview mainshock ruptured similar patches to the M4+ foreshocks. So both of the two previous M5+ mainshocks are likely related to earthquake-to-earthquake interactions. Does this mean that the Pawnee earthquake is special, and unexpected? In the next section, we show that the Pawnee earthquake does have small magnitude precursory activities leading up to the mainshock, which is only revealed with high resolution locations and a lower magnitude completeness.

## Results

### The mainshock triggering processes: precursory activities and injection

**Figure 4:**
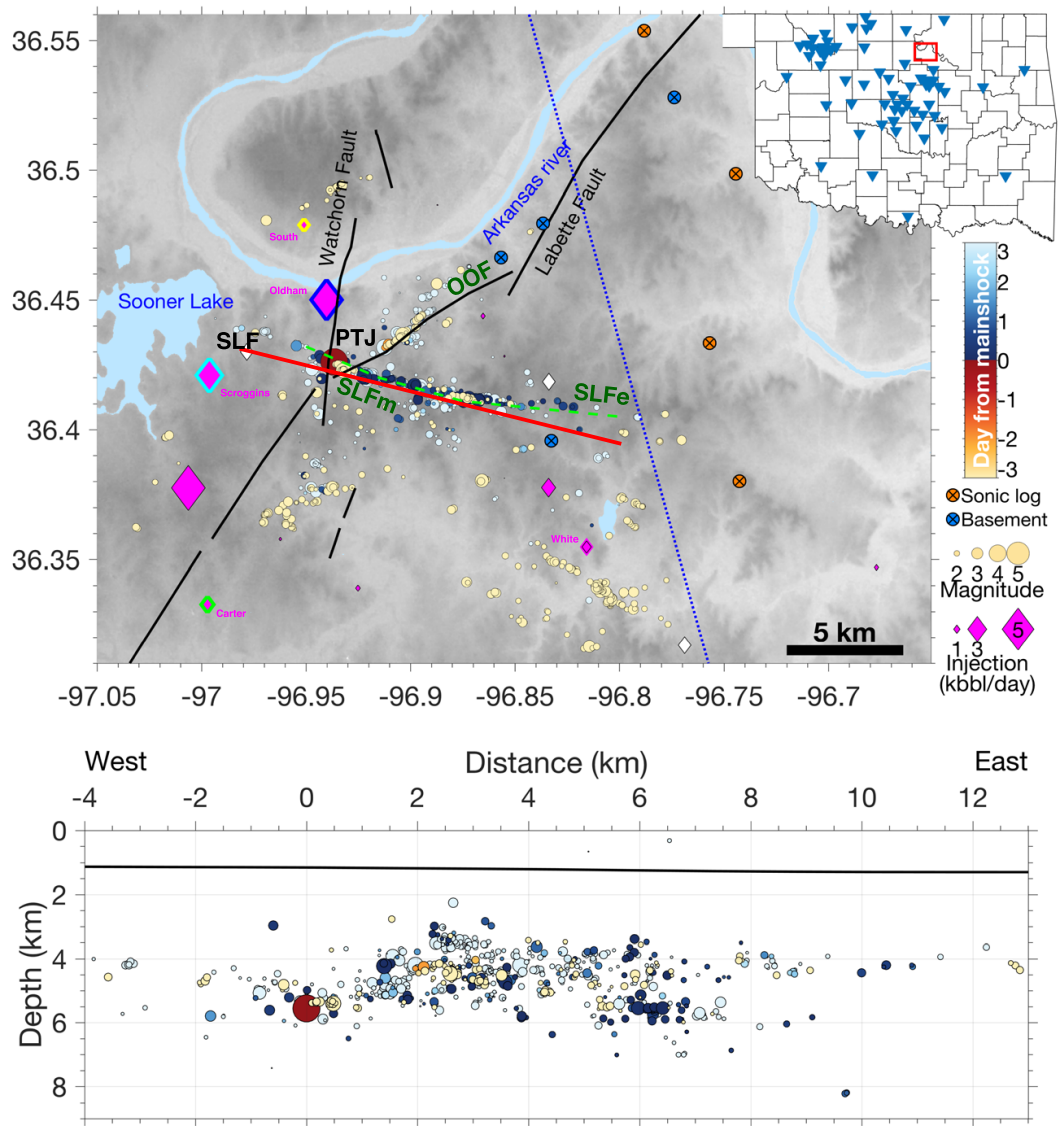
Top: Map view of the Pawnee earthquake region. Colored circles: relocated earthquakes in the study region since 2014. Magenta diamonds: disposal wells scaled by averaged injection volume since 2015. White diamonds: inactive disposal wells. Diamonds with colored outlines are wells shown in Fig. 6. The red line show the cross-section in the bottom figure, and the green dashed line shows the interpreted fault segments from earthquake relocation (SLFm and SLFe). Black lines show the mapped faults from OGS fault database^86^. Major geological structures are named on the map. “PTJ” refers to the intersect among multiple fault trends where the mainshock (the largest red circle) occurred. The blue dashed line shows the profile location in Figure S3, and the circles with “x” denotes wells used in Figure S3. Inset: the red box shows the magnified Pawnee region and the blue triangles are locations of the OGS seismometers on 2 September 2016. Bottom: Cross-section view of earthquakes within SLF along the direction of SLFm, the mainshock is located at distance 0. The averaged boundary between Arbuckle Group and basement is shown as a black line. The figure is generated with Matlab 2016a, available through: https://www.mathworks.com/.

The Pawnee earthquake struck at local time 7:02 AM on a Saturday morning, and widely felt throughout Oklahoma, and neighboring states (USGS “did you feel it” report). The earthquake occurred near the intersection of three fault traces (hereinafter referred as the Pawnee “triple-junction”: PTJ, see Fig. 4, with two mapped faults (Watchorn Fault and a segement of the Labette Fault) and the unmapped Sooner Lake Fault (SLF) where the mainshock ruptured and most early aftershocks occurred). The NE-SW trending mapped fault segment of the Labette Fault is optimally oriented in the present stress field (referred as OOF), and is regarded as a high-risk fault^37^. To better understand the seismicity evolution, we estimate precise locations for 950 earthquakes from 2014 to October 19th, 2016 based on double-difference algorithm combining analyst phase picks from the OGS, precisely measured differential-times via waveform cross-correlation and a 3D velocity model (see Method M5). The final catalog has a magnitude of completeness (Mc) of 2.0, and reveals several interesting observations. All the relocated earthquakes are located within the crystalline basement (the upper bound of the basement depth is between 1 km and 1.4 km) (Fig. 4), well below the depth interval of the Arbuckle Group where injection activities are occurring (Figure S3). We also use a catalog of 53 focal mechanism solutions from the OGS focal mechanism catalog to analyze seismogenic faulting.

#### Precursory activities suggest propagating aseismic slip

**Figure 5:**
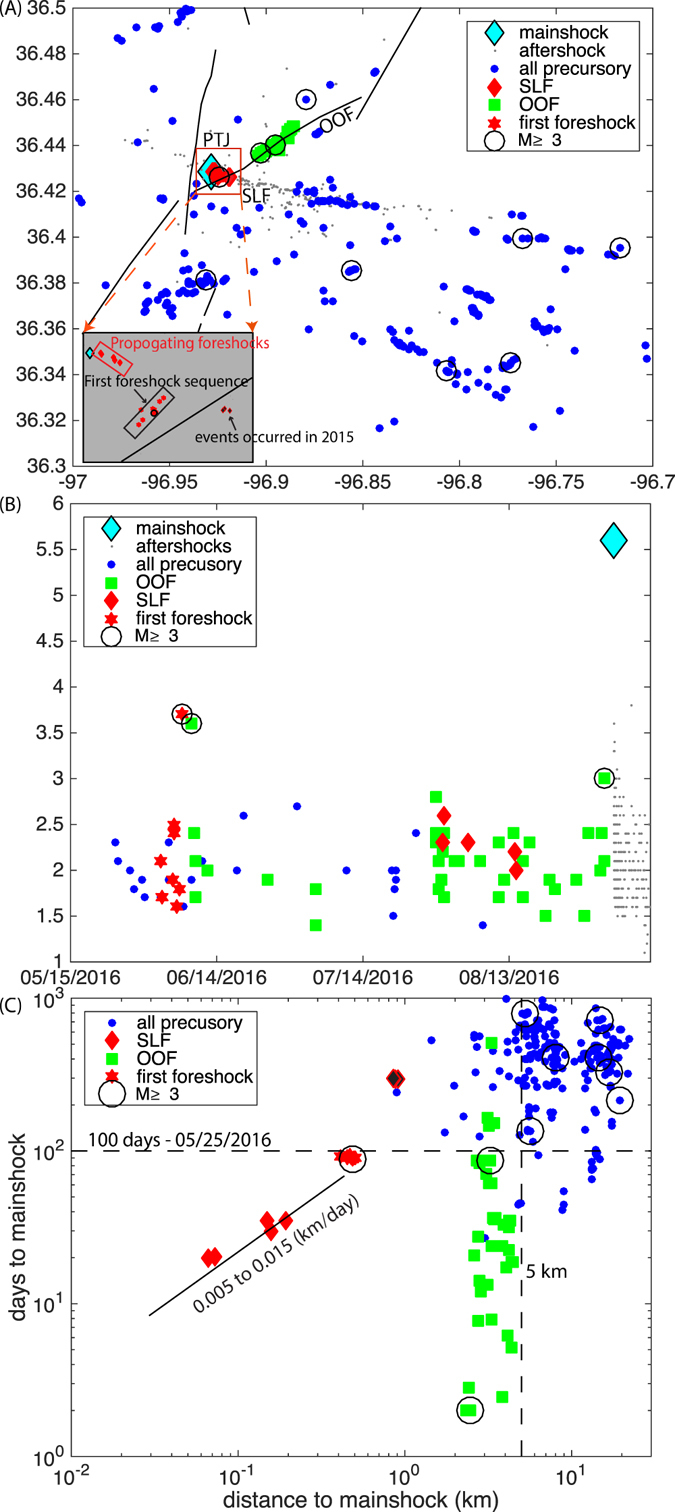
Foreshock migration leading to the mainshock. (**a**) Map view of foreshock and other background seismicity in this area. Earthquakes belong to different clusters are denoted with different symbols (see Map legend). The inset shows zoomed in view of the foreshock activities near PTJ, the first foreshock sequence and the foreshocks propagating to mainshock are enclosed in black and red boxes, respectively. Black lines show mapped faults, and black circles show events with M ≥3 that occurred before the mainshock. (**b**) Magnitude versus time for the 200 days period before mainshock. See legend for symbols. (**c**) Days to mainshock versus distance to mainshock. Note the apparent foreshock propagation within 0.5 km of the mainshock. The apparent migration velocity leading to the mainshock is between 0.005 to 0.15 km/day. The black diamonds with red outlines are three M1 events occurred west of OOF in 2015, which are not included in figure (**b**).

Key observations summarized from the high-precision catalog (from Figs 4 and 5):

- The early aftershocks delineate a conjugate fault to the OOF.
- The previously umappped fault (SLF) delineated by aftershocks changes strike from 107° (SLFm, where the mainshock occurred) to 98° (SLFe) at a structural bend (Fig. 4).
- All the three major seimogenic fault segments (OOF, SLFm and SLFe) are nearly vertical, with dipping angles of 89.7° from earthquake locations (Method M5).
- Approximately 100 days prior to the mainshock, two clusters with similar magnitude events (M3.7 and M3.6) occurred two days apart near the PTJ (on June 6th, 2016) and further north along the OOF (on June 8th, 2016) (Figs 5 and S4).
- Approximately 40 days prior the mainshock, two swarm-type clusters with mostly M2 earthquakes started two days apart (at OOF on July 28th, 2016, and at PTJ on July 30th, 2016) at similar locations as the previous two M3 events (Figs 5 and S4). The swarm along the OOF concluded with a M3 earthquake two days before the mainshock (Figs 5 and S4–c3).
- The foreshocks referred in this study only include the sequences within the conjugate fault system as described above, which are well separated from other earthquake clusters in this region, and concentrate within 5 km of the mainshock - the “Mogi” zone that is expected for large earthquakes^38^ (Fig. 5).
- The foreshocks within the PTJ are propagating towards the mainshock epicenter with propagation velocity between 0.005 and 0.015 km/day (Fig. 5, estimated from the linear trend of foreshock locations). While this migration velocity is lower than previously reported migration velocity for swarm activities elsewhere^39^, the apparent migration suggests possible existence of aseismic slip^40, 41^.
- While scattered activities occurred throughout 2015 in the study region (including SLFe, east of the structural “bend”), the SLFm mostly remained quiet at M2 level, except for the foreshock activities as described above (Fig. 5 and S4–c1).

Based on these observations, the near-simultaneous occurrence of clusters near PTJ and along OOF suggests possible Coulomb stress interaction within the conjugate fault system. However, Coulomb stress is considered a static effect, and may not well explain the apparent spatial migration pattern and the sequential occurrence of events. We interpret this as a result of possible precursory aseismic slip propagating within the mainshock nucleation zone, which drives the foreshock migration. Aseismic slip leading to large earthquakes have been widely reported for thrust faults in the subduction zones^13, 42^, and continental strike-slip faults^14^. Analysis of three M7 earthquakes in California found that they all have concentrations of small earthquake activities migrating in close proximity of the mainshock epicenter^17^, consistent with the observation for the Pawnee earthquake here. The previously reported aseismic processes mostly occurred at plate boundary faults naturally; however, some aseismic slip can be induced by fluid injection (e.g, refs 15, 16). This leads our next outstanding question: are the precursory episodes potentially related to injection?

#### Injection modulated precursory activities

**Figure 6:**
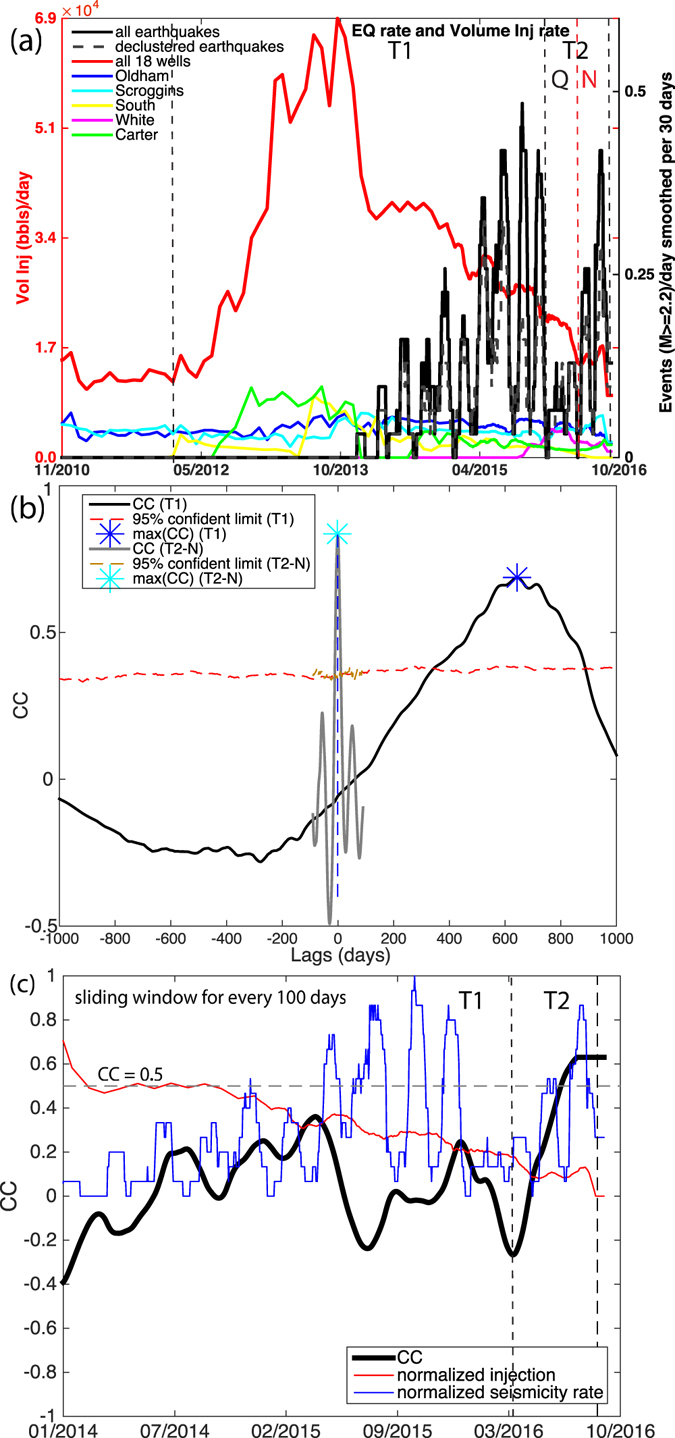
Relationship between injection and seismicity in the Pawnee area. (**a**) Black line: daily earthquake rate for *M* ≥ 2.2 events, smoothed for 30 days. Colored lines: daily injection volume for all 18 wells in Fig. 4 combined (red) and selected individual wells. Zoomed-in view of T2 is shown in Figure S8. (**b**) The cross-correlation between injection rate and seismicity for T1 (black solid line, with blue dashed line showing the maximum correlation), and for T2-N (grey solid line, with cyan dashed line showing the maximum correlation), the red and brown dashed lines show the 95% confidence limit. (**c**) Sliding time window correlation between smoothed injection and seismicity rate (thick black lines). The normalized injection and seismicity rates are shown as thin red and blue lines for reference. The dashed line denotes “cc = 0.5” to show periods with high correlation, which is only observed in period “T2”. This uses the relocated catalog.

We analyze the relationship between earthquakes from both the original catalog and declustered catalog with aftershock sequences removed^43, 44^ and injection records from 18 wells within 20 km radius around the Pawnee M5.8 mainshock. Similar to the statewide report (Fig. 2), the injection peaked in 2012 and 2013, then gradually decreased since 2014 (Fig. 6a). We divide the earthquake history into period T1 (from 2012 to May 2016) and T2 (from May 2016 to end of August: before the Pawnee M5.8) based on the timing of short period of seismic quiescence in early 2016 (see Fig. 6). In this section, we first analyze the two time periods separately with a 30-day smoothing window (as the injection rate prior to 2014 is only available in monthly report), then we apply a sliding-window analysis for the same smoothing window^44^; finally, we use a matched-filter completed catalog to examine the foreshock period in more detail with finer temporal resolution.

During period T1, both the earthquake rate and cumulative count of earthquakes resemble the shapes of injection rate and cumulative injection volume, respectively. The time delay for the declustered catalog is 643 days with maximum CC of 0.69 (Fig. 6a); the time delay for raw catalog is 717 days with maximum CC of 0.65. In both cases, the correlation significantly exceeds the 95% confidence limit; therefore, earthquakes during this period are most likely related to the peak injection of 2012 and 2013, which is consistent with the pressure modeling^45^. The difference in time delays observed from the statewide catalog and the local catalog within the Pawnee region is likely due to variations in hydraulic diffusivity, and average distance to injection wells, but the important observation here is that there exists a statistically significant correlation between injection rate and earthquake rate.

During period T2, this region is seismically inactive between January and May 2016 (T2-Q in Fig. 6 - “Q” for seismic quiescence). Starting in May 2016, seismicity rate increases again following small amplitude fluctuations in injection rate. The two peaks in seismicity rate correspond to the two M3.5+ earthquakes 100 days before, and two swarms 40 days before the M5.8 (see discussion in section). The time period between May 2016 and August 2016 is referred to as T2-N (“N” for mainshock nucleation). In contrast to the long time delay during period T1, seismicity rate is almost instantaneously responding to short-term fluctuations in injection rate: maximum CC of 0.81 occurs at 0-day time lag to the total injection rate with 30 day smoothing (Fig. 6b), suggesting that the foreshocks that are leading to mainshock rupture are likely related to injection.

To confirm that the observed time delay difference between T1 and T2 is not due to the difference in the length of the time windows used in cross-correlation analysis, we perform a sliding-window analysis (Method M3). Figure 6c shows that significant correlation (i.e., CC ≥0.5) is only observed during T2, but not during T1. This confirms the difference in time delay observed previously.

**Figure 7:**
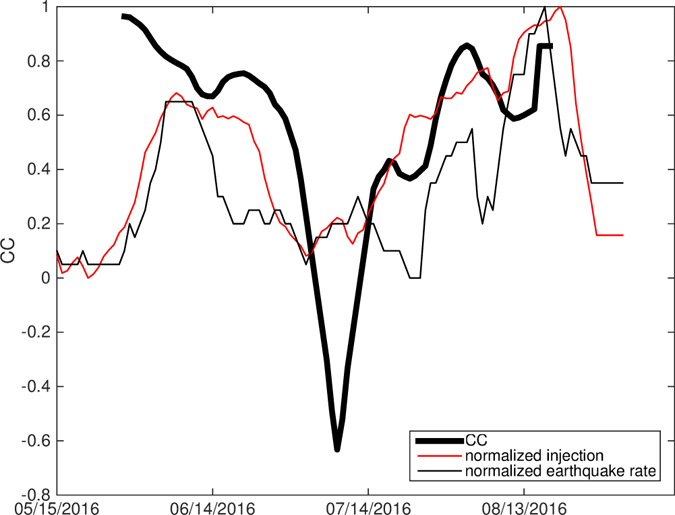
Sliding time window cross correlation (thick black line) between injection (red) and seismicity rate (black) from matched filter detection during the four months prior to mainshock. Both injection rates and seismicity rates are smoothed by 10 days, and the sliding time window is 30 days. A similar figure with 30 days smoothing and 60 days sliding window is shown in Figure S9, which shows more stable correlation. This uses the matched-filter completed catalog for higher temporal resolution.

To provide more detail of temporal variations during T2, we analyze a foreshock catalog obtained through matched-filter detection starting from April 1st, 2016 (Method M4)^46^. We apply a 30-day sliding-window analysis with 10-day smoothing window, and find that the two peaks in seismicity rate closely matches the peaks in injection rate with a short time delay (1–2 days) (Fig. 7). Different smoothing parameters all produce significantly high correlation between injection and seismicity at zero time lag (e.g., Figure S9 with 30-day smoothing and 60-day sliding window). We further analyze the time delay with the nearest two wells that dominate the temporal variations in injection rate during T2. The daily correlation is sensitive to a few days with “spiky” injection rate or earthquake rates. The time delay ranges from 2 days to 10 days, and is strongly influenced by a few spikes. Both the sliding-window analysis at different time scales and individual well analysis all find near-instantaneous responses to injection rate during T2, which is substantially shorter than T1, suggesting that this conjugate fault system is extremely sensitive to small stress perturbations prior to the mainshock rupture, revealing an extended nucleation process of the Pawnee mainshock. At 2.4 km distance (the approximate distance from well “Oldham” to mainshock), the expected time delay for pore pressure diffusion with diffusivity of 0.05 and 0.5 *m* ^2^/*s* would be 106 and 10.6 days. For coupled poroelastic stress, it allows rapid stress transfer through rock matrix and can have comparable or even larger amplitudes compared to pore pressure diffusion at shorter time delay^45^, ^47–51^. Therefore, the relatively shorter time delay would imply that some degree of elastic stress coupling was involved, however, the specific coupling would depend on local hydraulic parameters.

While a full scale reservoir modeling is beyond the scope of this paper, a pressure modeling demonstrated that the injection transient from two wells in 2012 and 2013 in the Osage county dominates the long-term stress changes on the mainshock fault^45^. The accumulated pore pressure amplitude is about a few tens of kPa from these two wells, and the coupled poroelastic stress is about a factor of two to three times larger at shorter time delay^45^.

During both T1 and T2, the declustered seismicity rate overlaps with the raw seismicity rate initially, but gradually deviates from the raw rate towards later stages for each period (Fig. 6b and S7). In earthquake triggering models, the declustered catalog reflects the “background” seismicity rate changes, which is directly linked to external stressing rate changes. The aftershocks that are removed from the declustering process are primarily due to “earthquake-to-earthquake” interaction, triggered by previous earthquakes due to cascading failure^4, 52^. The increasing deviation with time from the two catalog suggests that continuing weakening of the fault zone may facilitate earthquake-to-earthquake interaction, and that aftershock productivity may differ from natural earthquakes, e.g., the Arkansas induced earthquakes have higher productivity compared to a previous natural earthquake swarm^4^. Potential temporal changes in aftershock triggering parameters would warrant further study, and should be incorporated into hazard assessment for induced seismicity. This also indicates that in addition to the aseismic slip propagation, some degree of stress triggering due to foreshock themselves may exist, which we will investigate in the next section.

#### Coulomb stress and fault interaction

**Figure 8:**
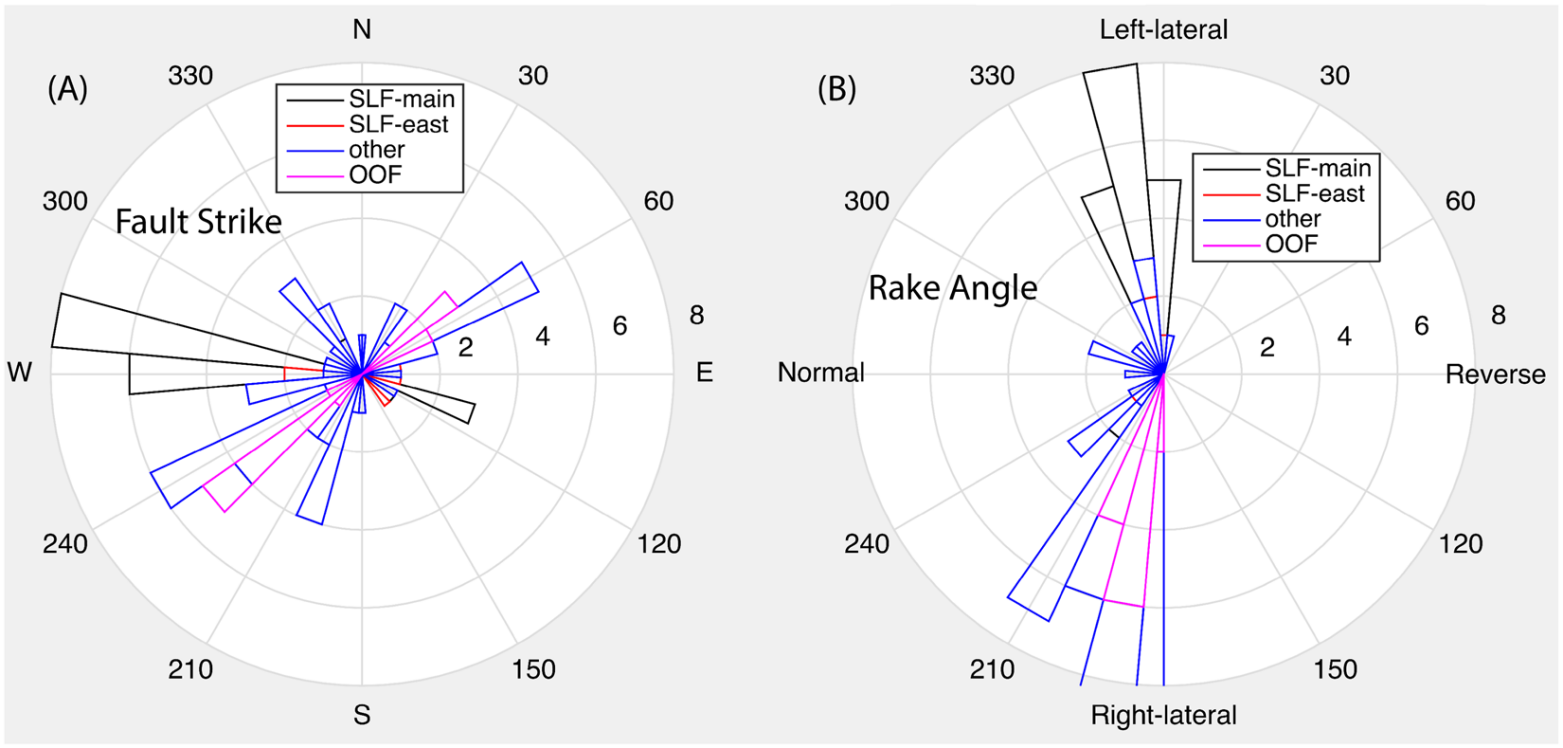
(**A**) Rose diagram of strike angles of nodal planes for individual earthquakes.For events on SLF-main and SLF-east, left-lateral nodal planes are selected. For events on the OOF, right-lateral nodal planes are selected. For other events, no preference is chosen. (**B**) Rose diagram of rake angles for the same set of earthquakes, which suggests a transtensional environment.

**Figure 9:**
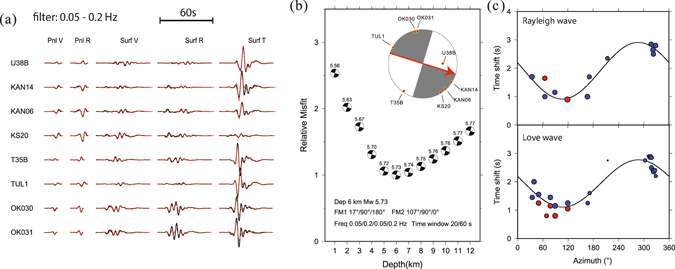
Source parameter inversion for the mainshock. (**a**) Waveform fitting in the CAP inversion. The black and red traces are observed and synthetic velocity seismograms, respectively. (**b**) Waveform misfit versus centroid depth. The number above the small beach ball is the moment magnitude. Stations used in the inversion are indicated as dots on the large beach ball. Red line in the beachball indicates the preferred nodal plane, and the arrow indicates preferred rupture direction. (**c**) Observed (dots) and best-fitting (lines) azimuthal variation of the time shifts for Rayleigh wave (top) and Love wave (bottom). Blue and red dots indicate observed time shift without and with calibrations of a reference event, respectively. The curves show the predicted time shifts with rupture along the nodal plane with a strike of 107°. The figures are generated from GMT, which is an open source software available at: http://gmt.soest.hawaii.edu.

For the Pawnee earthquake, SLFm had remained mostly locked throughout 2014 and 2015, while the surrounding areas have been active, including activities immediately east of the “bend” along SLFe (see Fig. 5). Based on fault plane solutions in this area (catalog obtained from OGS), this region is located within transtensional regime that is dominated by strike-slip faulting, with a few dip-slip events (Fig. 8). For the mainshock, we perform a waveform modeling using regional stations, and obtain two nodal planes with strike/dip/rake of 17°/90°/180° and 107°/90°/0°, respectively. The aftershock distributions and directivity analysis (Fig. 9c) indicate that the strike of 107° is the primary fault plane. Combining the fault plane solutions for all events in our catalog, we infer that the SLF is left-lateral strike-slip fault, and OOF is right-lateral strike-slip fault. The OOF is located within the step-over between two segments of the large regional Labette Fault. Fault orientations of other earthquakes that are located outside the conjugate fault system (including the OOF and SLF) appear much different (Fig. 8), suggesting that the activities within the conjugate system is likely different from other background activities, which further justifies the statement of foreshocks in previous sections.

**Figure 10:**
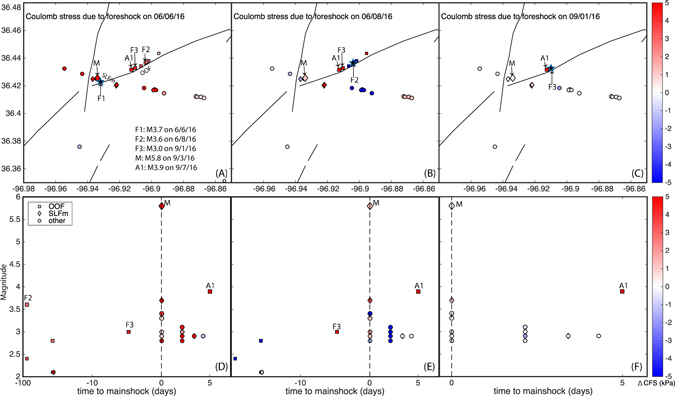
Coulomb stress change on foreshocks, mainshock and early aftershocks due to three M3+ foreshocks. Top row: Map view of Coulomb stress changes due to individual foreshocks. Bottom row: Magnitude versus time view of Coulomb stress changes due to individual foreshocks. The labels of are listed in Figure (**A**). The legend is shown in Figure (**D**). The “OOF” denotes for the conjugate mapped fault, and “SLFm” denotes the segment of Sooner Lake fault ruptured during the mainshock, see Fig. 4).

The earthquake clusters along OOF and near PTJ are nearly simultaneous both around 100 days and 40 days before mainshock (approximately two days apart) (Fig. 5), suggesting possible stress interaction within the fault system, well before the mainshock occurred. Along OOF, all the M3+ earthquakes occur within 1 km of each other: the M3.6 event in June 2016, the M3 earthquake two days before the mainshock, and a M3.9 aftershock 4 days after the mainshock (Fig. 10). Simultaneous slip (within hours or days) along conjugate faults has been observed at a variety of tectonic settings, for example, the 1987 Elmore Ranch/Superstition Hills earthquake pair were separated by one day (scedc.caltech.edu). Here, we perform detailed Coulomb stress analysis to assess the stress interaction between foreshocks and the mainshock.

The first M3.7 foreshock (denoted as F1) on June 6th is located at 0.3 km to the mainshock near PTJ. It is the last event of a five-day earthquake swarm that started following injection rate increase from a few nearby wells, including wells “Oldham” and “Scroggins” (Fig. 6). Three M2 earthquakes occurred prior to F1: M2.2 on 6/2, M2.3 and M2.5 on 6/5 (separated by 2 minutes). These three M2s all produced negative coulomb stress on F1, suggesting that the largest foreshock is not directly triggered by previous events. Rather, the strong correlation between foreshock episodes and injection in section suggests that the first foreshock sequence is likely induced by injection (Fig. 7). We then calculate the Coulomb stress changes by this foreshock on subsequent foreshocks and the mainshock (see Method M8). Figure 10A and D shows that this foreshock caused positive Coulomb stress changes on all the subsequent foreshocks and the mainshock, where the mainshock received Coulomb stress increase of 0.6 bars (60 kPa) with friction coefficient of 0.4^53^. Given that this foreshock produced positive Coulomb stress on all subsequent foreshocks, it should also contribute to the triggering of the aseismic slip episode that drives the migration of foreshocks within mainshock nucleation zone, although these events were too small to have well-constrained focal mechanism solutions.

The M3.6 foreshock on June 8th, 2016 (denoted as F2) produced Coulomb stress increase on the next M3 foreshock, and the M3.9 aftershock that occurred 4 days following the Pawnee mainshock. Event F2 triggered its own aftershock sequences on OOF. The M3 foreshock on September 1st, 2016 (denoted as F3) also promoted failure for the M3.9 aftershock. Both of these two foreshocks are located much further from the mainshock, and cause small but positive Coulomb stress changes at the mainshock (mainly due to changes in normal stress)^53^.

The Coulomb stress analysis shows that all the foreshocks contributed to the triggering of the mainshock, and the first foreshock promoted cascading failure for all subsequent foreshocks for which we have focal mechanism solutions, and some early aftershocks. However, this event itself was not triggered by previous events in the same cluster, and is likely due to injection rate increases. The other two M3+ foreshocks contributed to triggering of other foreshocks and early aftershocks.

## Discussion

### Triggering process of the Pawnee mainshock

Combining the analysis of seismicity, injection, and stress interaction, we speculate the following scenario for the Pawnee earthquake occurrence:

- The SLFm is a strong blind fault that can sustain the high pore pressure throughout the high injection period;
- All the seismogenic fault segments in this region (SLFm, SLFe and OOF) are critically stressed with continuing pore pressure increase due to long-term fluid diffusion and ready to slip with small stress perturbations;
- After a short period of quiescence in early 2016, operation from a few nearby wells led to small increases in injection rate, followed by rapid seismic responses on the conjugate fault system, and triggering of the first foreshock sequence, including a M3.7 earthquake near PTJ;
- The M3.7 foreshock caused Coulomb stress increase on most subsequent foreshocks and the mainshock, and promoted fault slip within the conjugate fault system, including a M3.6 earthquake along OOF, which also promoted failure for the next M3 foreshock, and a M3.9 a cks very well, and is subject to severer clipping problems ftershock along the same fault segment (Fig. 10);
- The second stage of injection rate increase correlates with second stage of foreshock activities, which includes foreshocks near PTJ that appear to be migrating within 0.5 km of the mainshock, suggesting a possible aseismic slip episode - it should be noted that although the foreshocks appear migrating towards the mainshock, the relative locations between the foreshock sequence and the mainshock may subject to greater location uncertainty than within the foreshock themselves, as the waveform of the large mainshock does not correlate with the smaller foreshocks very well, and is subject to severer clipping problems due to strong ground motion^17^, ^54^;
- The second stage of foreshock activities also includes a second earthquake swarm along OOF, which culminated with a M3.0 earthquake just two days before the mainshock. The M3 event promoted failure for a M3.9 aftershock following the mainshock, and small positive Coulomb stress on the mainshock.
- The Coulomb stress on the mainshock from the first foreshock is significantly larger than the two later foreshocks (60 kPa versus about 0.5 kPa), likely due to its closer distance to mainshock (0.3 km versus 2 km).
- The aseismic slip propagation, stress loading from the foreshock activities, and the injection continues to increase stress in the mainshock nucleation zone, and eventually triggered the mainshock rupture.

Once the rupture initiates and propagates along the fault, the eventual size of the mainshock is limited by the physical properties of the fault zone and the earthquake rupture itself, as any earthquakes, regardless of the eventual magnitude, grows from a small rupture. Analysis of different sized earthquakes in Parkfield, found that the first 0.03 seconds of the rupture are indistinguishable for different magnitude events^55^. The physical parameters that determine the size of an earthquake are the macroscopic properties of earthquakes, such as the rupture size, duration, and total amount of slip^56^. Among them, the rupture size is limited both by the physical length of the fault and the rupture front propagation: larger faults are capable of generating larger earthquakes, e.g., the San Andreas Fault, and the Meers Fault; and the capability of the rupture front propagation along the fault, which is related to the dynamic interaction between the stress of rupture front and the friction parameters (e.g., “strengthening” of rupture front would tend to inhibit rupture growth, while “weakening” would facilitate rupture growth)^57–59^.

The estimated rupture length from the directivity analysis is about 6 km (Method M7) assuming a unilateral rupture along 107°, which is consistent with a recent finite slip model of the Pawnee earthquake^60^. The estimated rupture extends about 6 km to the east from the mainshock epicenter, which roughly corresponds to the distance from the mainshock epicenter to the structural “bend” between SLFm and SLFe. It is possible that the structural complexity may serve as a “barrier” to the mainshock rupture. Further aftershock analysis may better constrain the faulting complexity.

### Best way forward

The overview of earthquakes and injection well distributions in Oklahoma suggest that the spatial distributions of the M5+ earthquakes are controlled by regional tectonic faults. Detailed analysis of the Pawnee earthquake precursory activities shows that the nucleation process of the Pawnee mainshock is a result of the interplay among injection rate increase, earthquake interactions and aseismic slip. Will the mainshock occur without either process? Which process is more dominate in the triggering process? These questions are difficult to address given the simplified model assumptions in a heterogeneous environment. It appears that Coulomb stress from the M3.7 foreshock (about 60 kPa) is comparable to or larger than the injection induced stress changes from long-term injection^45^, but would depend on specific model assumptions in both Coulomb stress calculation and pore pressure modeling. The aseismic slip induced stress amplitude is difficult to assess due to the lack of absolute slip rates, however, it has been suggested to play an important role in triggering large plate boundary earthquakes, e.g., subduction zones^13^. The rapid response to small perturbations in injection rates during T2-N suggests that the fault is every sensitive to external stress perturbations, and would probably be triggered by any process that causes stress changes. Similar to the Prague M5.7 mainshock that was likely triggered by the M5 foreshock^12^, the Pawnee earthquake was plausibly triggered by precursory seismic and aseismic slips on the conjugate fault system. In both cases, the mainshocks ruptured previously unmapped basement faults, for which prior earthquake hazard assessment was not possible.

The small magnitudes of the precursory activities for the Pawnee earthquake highlights the importance of high-resolution monitoring of microearthquakes. In this case, the “foreshocks” and the changes in the response to injection rate during T2-N would not have been recognized if we only focused on M3+ earthquakes and did not have a high resolution relocated catalog. As most of the high-resolution relocation is usually performed retrospectively, it is challenging to recognize the spatial-temporal pattern in real-time.

However, even assuming that all the relocation and detections can be done perfectly in real time, it is still challenging to separate “foreshocks” from a randomly occurring earthquake swarm. In most cases, “foreshocks” are recognized only after the larger event, except the rare case of a successful earthquake prediction for the Haicheng earthquake in 1976^61^. Systemic analysis of foreshock occurrence patterns in California for *M* ≥ 5 earthquakes suggests that an important diagnostic feature of foreshocks is the increased seismic activities within the “Mogi” zone of a large seismogenic fault, especially those that are associated with fault zone discontinuities^62^. The “Mogi” zone is defined as a seismic quiet zone surrounding the mainshock, within a radius that is approximately consistent with mainshock rupture length^38, 63^. So for an M6 earthquake, this would be about 5 km to 7 km. In this case, most of SLFm has been quiet (at least 6 km in length), except the 0.5 km area immediately surrounding the mainshock, well within the expected “Mogi” zone (Fig. 5). In addition, it is located within structural complicated area with intersections of several faults, consistent with the observations in California for the three M7 earthquakes where all foreshocks localize in areas with structural complexity^17^. With regard to spatial clustering, the nucleation process for the Pawnee earthquake is similar to tectonic earthquakes.

In a region with dominantly “swarm-type” clusters, or most mainshocks have foreshocks (both are consistent with cases in Oklahoma), increased “swarm” activity near major fault segments may suggest an increased probability of a larger earthquake (e.g., Landers and Hector Mine earthquakes)^17^. However, in this case, it would have been challenging as the SLF was previously undocumented.

Thus, the detailed understanding of earthquake triggering processes and possible early recognition of damaging earthquakes would require high-quality fault map and high-resolution monitoring of microearthquakes, preferably down to much lower magnitude level^64–67^ with much higher location resolution via waveform cross-correlation^54, 68, 69^. The observation of potential aseismic slip leading to the mainshock suggest that geomechanical analysis of the frictional behaviors of the fault zone would be very important to better understand the mainshock rupture process^70^. The rapid response to injection rates indicates a possible role of poroelastic stress responses: if this proves to be significant with further detailed reservoir modeling, it would suggest that we should probably consider injection to all formations in addition to Arbuckle Group for specific study areas. The detailed analysis of precursory activities for the largest earthquake in Oklahoma suggests that we need to look beyond fluid diffusion, and consider the full spectrum of triggering processes, fault slip, and stress transfer mechanisms to obtain a complete view of the nucleation process of large earthquakes. Partnership between industry, government and academia to investigate the cause and mitigation of the potential earthquake hazards will be the best way forward with mutual understanding of the importance of a fault database, pressure monitoring and earthquake monitoring.

## Methods

The method section includes:

- M1: Spatial density analysis of events and injection rates
- M2: b-value estimation
- M3: Calculation of correlation coefficients and confidence limits
- M4: Matched filter detection
- M5: Earthquake relocation and fault parameters
- M6: Catalog declustering
- M7: Waveform inversion using CAP method
- M8: Coulomb stress analysis

### M1 Spatial density analysis of events and injection rates

We use the earthquake catalog from the Oklahoma Geological Survey (OGS). All earthquakes between 2011 and 2016 are spatially binned in a 0.025° latitude/longitude (approximately 3.6 km) grid. For each grid, the number of events above magnitude 2.5, 3, 3.5, 4, 4.5, and 5 were counted. To obtain the final image, we apply a Gaussian smoothing filter within a square area of 5 grids for each grid (approximately 18 km). For each magnitude range, the average number of events for all grids with seismicity is selected and contoured (Figure S1). Each average contour is then plotted together in Fig. 1 to show the location of most events within each given magnitude range.

For injection data, we organize a complete database for all wells injecting into the Arbuckle Group since 2011. The wells are spatially binned in the same 0.025° latitude/longitude grid as earthquakes. For each grid, the average daily injection rate between 2011 and 2016 is calculated. Similar Gaussian smoothing filter is applied to all grids with non-zero injection rates. The average injection rate for grids with active injection are selected and contoured. The contour line is plotted together with the contour lines for each magnitude range in Fig. 1 to compare with earthquake distributions.

### M2 b-value estimation

For b-value and Mc estimations, we use the software “zmap” subroutines^71^. The Mc is estimated through maximum curvature in the histogram for earthquake number per magnitude bin. The bin size is 0.1. The b-value estimation follows the maximum likelihood method^72^:

3 $$b={\mathrm{log}}_{10}e/(\bar{M}-{M}_{min})$$

For the statewide analysis, we use *M*_*min*_ of 3 (Fig. 3), and for the Pawnee aftershock, we use *M*_*min*_ of 2.2 (Figure S2).

### M3 Calculation of correlation coefficients and confidence limits

For the two time series injection (I) and seismicity (S), we compute the normalized correlation coefficients for each time lag following Matlab function “crosscorr”. At each time lag *k*, the cross-covariance is:

4 $${C}_{I,S}(k)=\{\begin{array}{ll}\frac{1}{N}\sum _{j=1}^{N-k}({I}_{j}-\bar{I})({S}_{j+k}-\bar{S}); & {\rm{k}}=0,1,2,\mathrm{..}.\\ \frac{1}{N}\sum _{j=1}^{N+k}({I}_{j-k}-\bar{I})({S}_{j}-\bar{S}); & {\rm{k}}=0,-1,-2,\mathrm{..}.\end{array}$$

where $\bar{I}$ and $\bar{S}$ are the sample means of the series. The auto-correlation for each series is:

5 $$\{\begin{array}{rcl}{A}_{I} & = & \sqrt{{C}_{I,I}\mathrm{(0)}}\,{\rm{for}}\,{\rm{injection}}\\ {A}_{S} & = & \sqrt{{C}_{S,S}\mathrm{(0)}}\,{\rm{for}}\,{\rm{seismicity}}\end{array}$$

The normalized cross-correlation is:

6 $$C{C}_{I,S}(k)=\frac{{C}_{I,S}(k)}{{A}_{I}{A}_{S}};{\rm{k}}=0,\pm 1,\pm 2,\mathrm{..}.$$

To estimate confident limit, we assign random phase shift for each frequency for the Fourier transform for each series, so its auto-correlation is unaffected^73^. 1000 cross-correlations are obtained with each assigning different random phase shifts. The 95% confident limit is obtained from the 95% percentile limit at each time lag. All the maximum correlation coefficients at preferred time lags for both the statewide analysis and Pawnee county at different tiem periods are significantly above the 95% confident limit, suggesting that the correlation is not by random chance (see Figs 2b and 6).

To perform the sliding-window analysis to identify short-term correlations, we preset a fixed window length (*T*_*w*_), then for each day (*T*_*i*_) during the study period, we calculate the correlation coefficient using “corrcoef” function in matlab with injection and seismicity rate between *T*_*i*_ − *T*_*w*_/2 and *T*_*i*_ + *T*_*w*_/2. Because the “corrcoef” function would produce positive correlation between low injection rate and low seismicity rate (which may confuse the interpretation to search for positive correlations between increased injection rate and seismicity rate), we further calculate moving-average of the correlation results using the same window length *T*_*w*_. Figs 7 and S9 show results with different window options, and both suggest strong instantaneous response to injection rate changes. In Fig. 7, the normalization of both injection rate and seismicity rate is done by subtracting the minimum value in the time series, then dividing by the maximum value.

### M4 Matched filter detection

We downloaded regional seismic data throughout Oklahoma, including regional and temporary stations deployed by the Oklahoma Geological Survey and other agencies in the years prior to the Pawnee earthquake^46^. We manually picked aftershocks of the Pawnee mainshock, in order to focus on events that may be have repeated along the fault that eventually ruptured. Using Antelope’s *dbpick* waveform viewer, we picked phases and performed an initial location with *dbloc2*, utilizing a local tomographic 1-D velocity model from a previous study^26^.

Based on prior experience with regional network cross-correlation^67, 74^, we bandpass filter the templates between 4 and 10 Hz, cut the waveforms 1 s before and 10 s after the phase arrival (P or S), and resample the data at a 20 Hz uniform sample rate. The matched-filter technique computes the normalized cross-correlation coefficient (coefficient between −1 and 1) at each sample point for each individual template. We then shift each of the normalized cross-correlation functions for each individual component relative to the traveltime of each component and stack. Initial detection occurs when any point within the stacked cross-correlation function exceeds at least 9 times the median absolute deviation (MAD) of the daily stack^46^.

### M5 Earthquake relocation and fault parameters

312 earthquakes in our study area (last accessed: 10/19/2016) have analyst phase picks. To relocate all events systemically, we measure precise differential times through waveform cross-correlation between each event and all other events. We first compute predicted arrival time at each station for each event using 1D travel time table based on statewide 1D velocity model^75^. Then, we apply an auto-picker^76^ to pick around the predicted time. Only new picks with signal-to-noise ratio greater than 2.5 are saved for further use. All the traces band-pass filtered between 1 and 10 Hz. To measure cross-correlation, we select a time window 0.5 sec before and 1 sec after the picked arrival time (or predicted arrival time if neither analyst picks nor auto-picks are available) for both P and S waves. The final differential time has 0.001 second accuracy with sub-sampling around the peak correlation, and only pairs with CC ≥0.6 from at least four stations are used for relocation.

In total, we obtain 1,566,713 P wave differential times from cross-correlation, and 50,628 from catalog, 972,388 S wave differential times from cross-correlation, and 50,394 from catalog. Overall, the amount of cross-correlation derived differential times is a factor of 25 larger than catalog. For relocation, we use the double-difference algorithm, with the hypoDD3D version^77^ that incorporates 3D ray-tracing from a 3D tomography model^22^. This allows us to obtain precise locations for 950 earthquakes.

To estimate location uncertainty, we apply a bootstrap approach. We use the final locations from the relocated catalog as input. Then we generate 50 sets of arrival times with 80% of randomly selected cross-correlation and catalog differential times. We run the same program for all resampled datasets. The final location error for each event is set to the 95% of the location differences between resampled dataset and the original catalog, following standard approach to estimate 95% confidence limit for each event. The relative location accuracy is estimated to be 35 m horizontally and 200 m vertically (Figure S10). The vertical error is much greater than the horizontal error. For comparison, we also estimate individual location errors using the standard deviation of location changes. The resulted location errors are much smaller than the 95% confident limit (Figure S10). Since the spatial migration in Fig. 5 is focused on horizontal distances, the location precision is sufficient to show the spatial migration.

To estimate the strike and dip angles from the seismicity cloud, we use the approach from^78, 79^, and references therein, where we find the eigenvalues and eigenvectors for the covariance matrix of demeaned hypocentral coordinates of the events along each fault segment. The eigenvector of the largest eigenvalue defines the longest axis of an ellipsoid fit to the epicenters, while the eigenvector of the smallest eigenvalue defines the shortest axis of the ellipsoid. The strike and dip angles are found from the orientation of the largest eigenvector. All the three faults are nearly vertical with dipping angles close to 90°, consistent with the mainshock nodal plane solutions from waveform fitting (Fig. 9).

### M6 Catalog declustering

We use the declustering subroutine from the “zmap” software package^71^, and used the default parameters^43^. This method first models the spatial and temporal extent for each earthquake based on empirical relationships of the source dimension and temporal rate decay. Each earthquake is linked with other earthquakes if they are within the space and time window defined by the empirical relationships. Linked events will form a cluster, with the largest event in each cluster considered as the mainshock. With this method, the majority of the aftershocks from the Pawnee mainshock have been removed. The seismicity rate in the declustered catalog is substantially lower compared to the original catalog (Fig. 6), and reflect the variations of “background” seismicity rate that is likely directly linked to injection.

### M7 Rupture directivity and fault plane solutions

To model the source mechanism and depth, we collect broadband seismograms of 8 regional stations with good azimuth distribution from IRIS Data Management Center (IRIS-DMC), and apply the Cut-And-Paste (CAP) technique^80^. After removing the mean value, linear trend, and instrument response for each station, we rotate the three-component velocity waveforms to radial, tangential, and vertical components. We then grid search focal mechanism (strike, dip, and rake), focal depth, and moment magnitude to best fit the waveforms bandpass filtered between 5 s and 20 s. We use the Frequency-Wavenumber (FK) method^81^ to calculate the Green’s functions with the CUS velocity model. To accommodate inaccuracies in the 1-D velocity model, we also allow the Pnl waves and the surface waves to shift with respect to each other^80^. The time windows for Pnl part and surface waves are 20 s and 60 s, respectively. And the frequency range is 0.2–0.05 Hz (5–20 s) for both Pnl and surface waves. The inversion results show that the mainshock is a strike-slip event with a moment magnitude of 5.73, and a depth of 6 km (Fig. 1a,b). The two nodal planes are: 17°/90°/180° (NP 1), and 107°/90°/0° (NP 2), roughly consistent with the SLU moment tensor solution (195°/90°/180° for NP 1), the USGS body wave moment tensor solution (193°/85°/176° for NP 1), the USGS W-phase moment tensor solutioni (194°/74°/161° for NP 1) and the GCMT solution (19°/83°/−169° for NP 1). However, the centroid depths of different solutions vary from 2 km (USGS body-wave solution), 11.5 km (USGS W-phase solution) to 19 km (GCMT solution). Thus, we calculate teleseismic P wave synthetics using the CUS at the source side^82^ for 5 stations with high signal-to-noise ratio, and search for the best centroid depth with focal mechanism fixed as above. The waveform comparison for teleseismic P wave suggests a centroid depth of 7 km (Figure S11), consistent with the CAP solution (6 km), the SLU solution (8 km) and the relocations from arrival times and 3D velocity model (6.3 km).

To estimate rupture directivity, we apply the relative centroid location method^83, 84^. The method determines ruptured fault plane via measuring the difference between centroid and hypocenter location, which are straightforwardly derived from the time shifts in the CAP source inversion of the mainshock and a reference earthquake. We first determine the relative hypocentral location of the mainshock with repsect to a M4 event, then perform source parametrer inversion for both earthquakes. We test the cases of rupture along either strike, and the time shift against azimuthal variation can be fitted with a cosine curve, which indicates the rupture direction and rupture length. Both Rayleigh wave and Love wave suggest rupture to southeast along the 107° strike, and the estimated rupture length is 6.2 km and 5.8 km, respectively (Fig. 9). As unilateral rupture is assumed in this method, the inferred rupture length is lower bound, but is approximately consistent with distance from mainshock to the intersection between SLFm and SLFe (Fig. 4), suggesting that the structural complexity may act as a “barrier” to earthquake rupture.

### M8 Coulomb stress analysis

For Coulomb stress analysis, we use the software Coulomb 3.4^85^, available from USGS website. The software implements an elastic half-space model to calculate the stress changes in space and time. In this study, we use individual nodal planes as receiver faults for each earthquake that has focal mechanism solution^53^. For each foreshock, we use the default setting to calculate length and width for “strike-slip” faults with the corresponding magnitude. We assume that the location from the relocated catalog is the center of the fault plane. The friction coefficient is assumed to be 0.4, consistent with analysis of the Prague sequence that considers the effect of pore fluid pressure by assuming Skempton’s coefficient of 0.75^12, 53^.

## Electronic supplementary material

Injection wells and earthquakes

supplemental material

relocated earthquakes
