# Supplementary material for: The Pawnee earthquake as a result of the interplay among injection, faults and foreshocks
Source: Sci Rep. 2017 Jul 10;7:4945. doi: 10.1038/s41598-017-04992-z (PMC5504070; doi:10.1038/s41598-017-04992-z)
Supplement: Supplementary file 2 — supplemental material [file 41598_2017_4992_MOESM2_ESM.pdf]

The Pawnee earthquake as a result of the interplay among injection, faults and foreshocks

Xiaowei Chen[1,*], Nori Nakata[1], Colin Pennington[1], Jackson Haffener[1], Jefferson C. Chang[1,2], Xiaohui He[3], Zhongwen Zhan[4], Sidao Ni[5], and Jacob I. Walter[6]

[1]ConocoPhillips School of Geology and Geophysics, the University of Oklahoma, Norman, OK, USA
[2]Oklahoma Geological Survey, the University of Oklahoma, Norman, OK, USA
[3]University of Science and Technology of China, Hefei, China
[4]Department of Geological Science, Caltech, Pasadena, USA
[5]State Key Laboratory of Geodesy and Earth's Dynamics, Institute of Geodesy and Geophysics, Chinese Academy of Science, China
[*]Correspondence author: xiaowei.chen@ou.edu

ABSTRACT

This is the supplemental materials for the main paper, which are necessary to understand the background geological information, the details of data processing and zoomed in view of the precursory periods.

List of supplemental materials

- Additional figures associated with method and data processing in this document.
- Relocated earthquake catalog from 2014 to October 19, 2016 as a separate csv file. The format of the catalog is as follows: year, month, day, hour, minutes, seconds, latitude, longitude, depth, magnitude.
- Movie as a separate file showing injection volume changes and seismicity in the study area. The movie symbols are consistent with Figures S5 and S6.

References

1. Greig, P. B. Geology of Pawnee County, Oklahoma. *Oklahoma Geological Survey Bulletin 83* (1959).

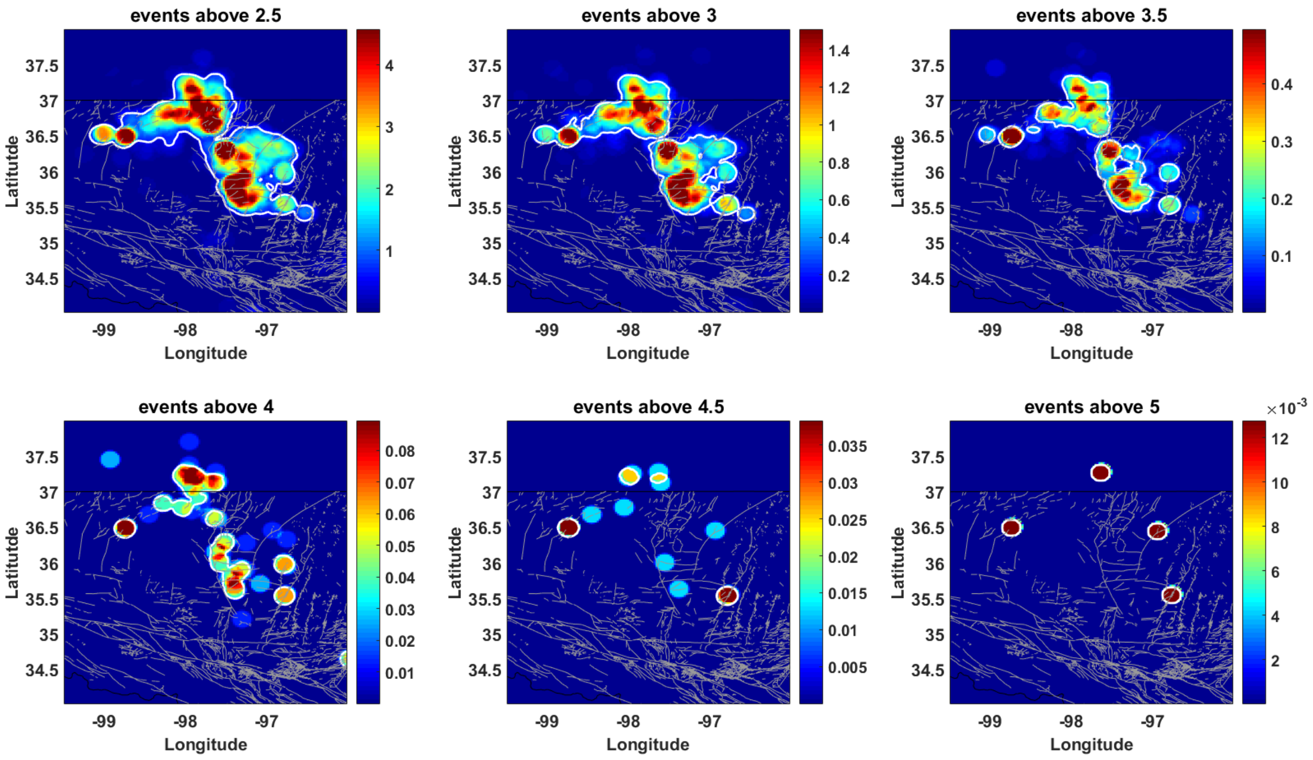

Figure S1. Smoothed density plot of events above a given magnitude for Oklahoma and southern Kansas. The white line denotes the average number of smoothed events for the given magnitude threshold and encircles the regions of dense seismicity for that range. The white lines are plotted together in Figure 1 for comparison (note, in Figure 1, only the Oklahoma side is shown).

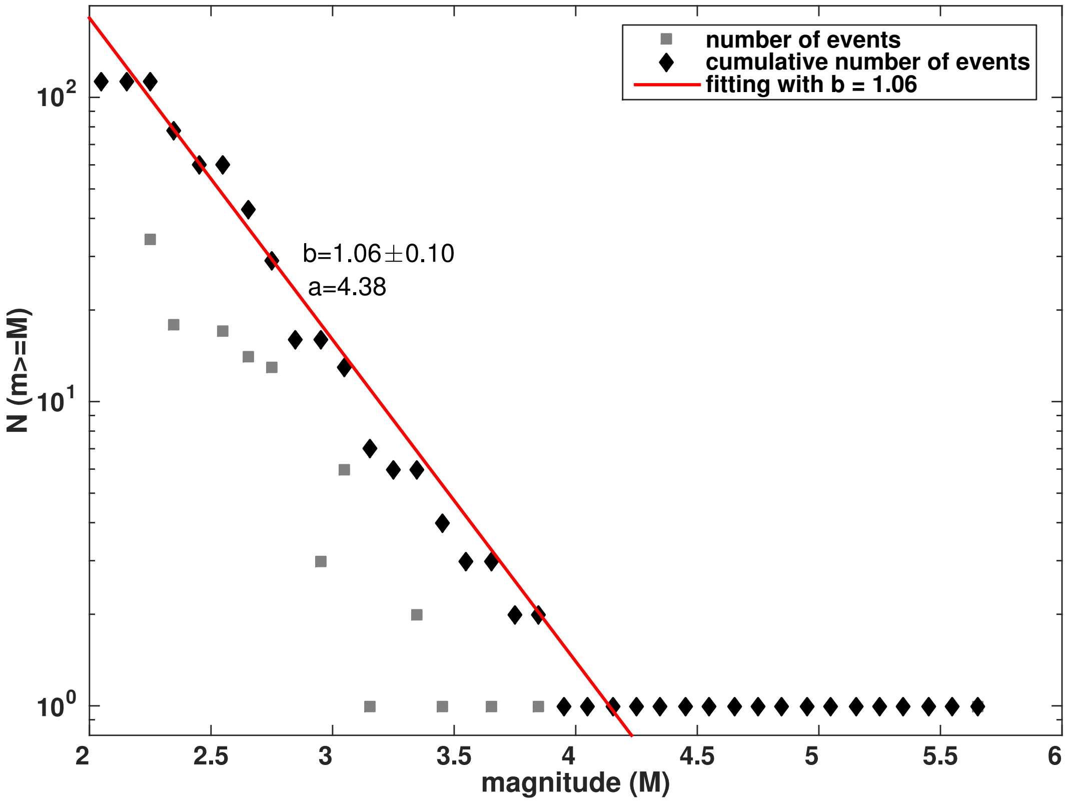

Figure S2. Magnitude-frequency relationship for the Pawnee sequence between the mainshock and Sep 14, 2016. The grey squares are number of earthquakes per magnitude bin, and the black diamonds are cumulative number of events per magnitude - N(m$\geq M$). Red line is the best-fitting with b-value of 1.06, and a-value of 4.38.

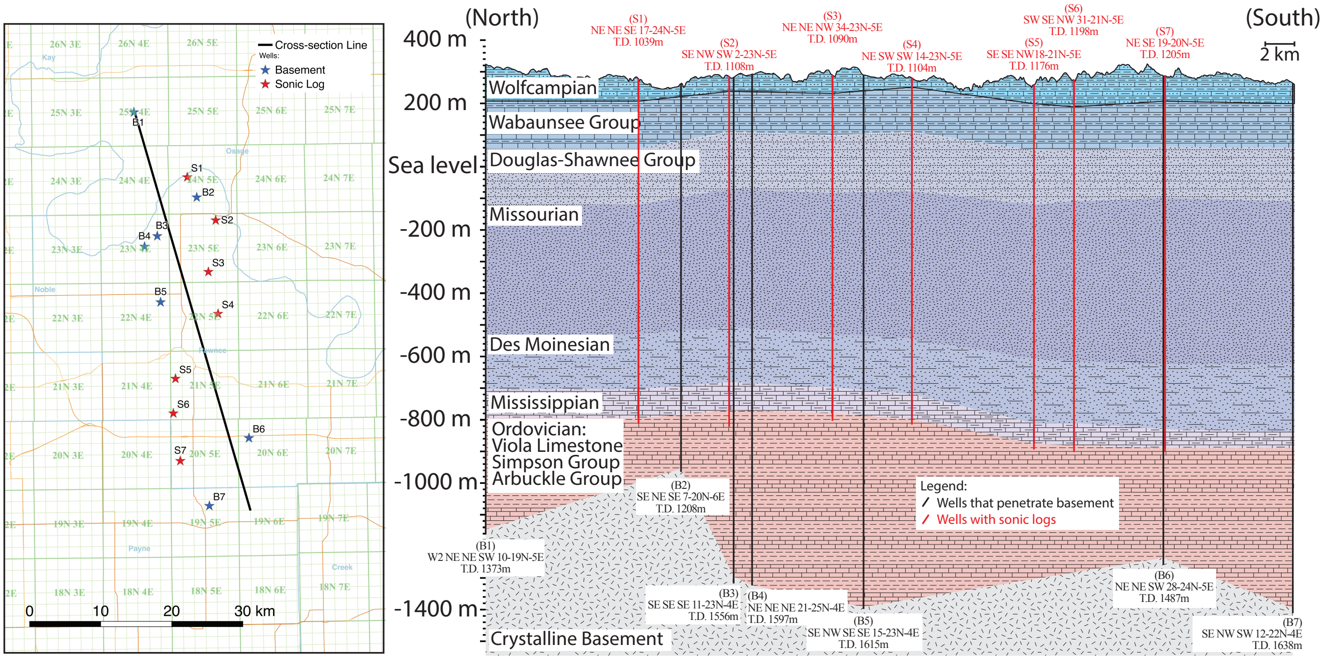

Figure S3. Left: Map view of well locations. The blue thin line denotes the Arkansas River. Right: Lithology along the profile. The sonic well logs and interpretation were published in an OGS Bulletin[1]. The basement penetrating well data are from an Oklahoma Petroleum Information Center (OPIC) dataset. The lithology depth constraints were taken directly from the well data, with straight line interpolation to depict the layers. It is important to note that the basement was subareally exposed during the deposition of the Arbuckle-Timbered Hills Groups, and the variations in basement depths shown are most likely due to paleo-topography from erosion rather than structural displacement.

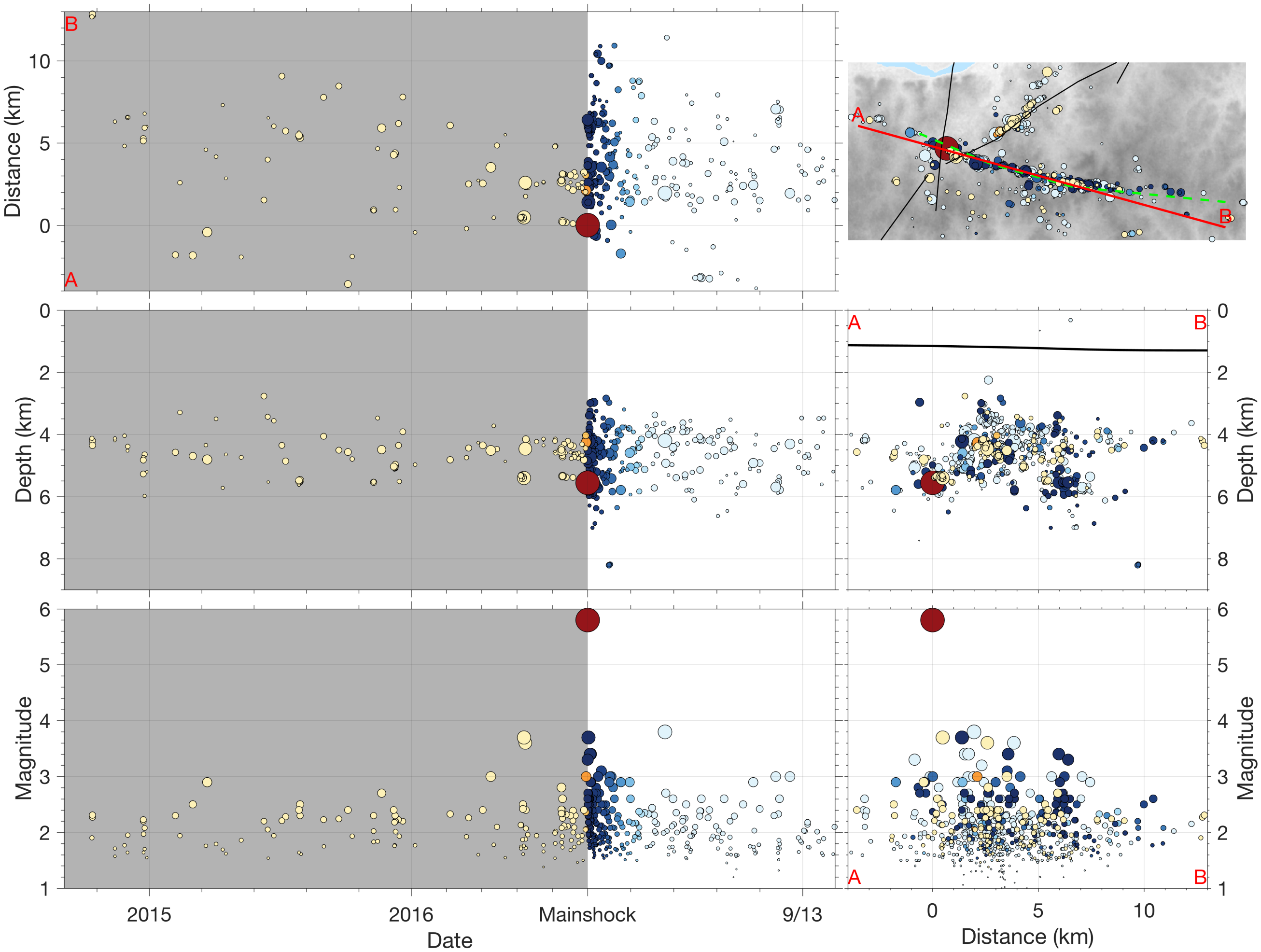

Figure S4. Overview of space and time evolution of seismicity. (a) Map view of seismicity (colored circles, same color scale as Figure 4), interpreted faults (green dash line), mapped faults (black lines), and profile for cross-section view (red line, from A to B). (b1) Cross-section view with distance along A-B versus depth. (b2) Magnitude versus distance along A-B. For figures (c1) to (c3), time before mainshock is shown in grey background, time after mainshock is in white with exaggeration of the time scale for display purpose. (c1) Distance along A-B profile versus time. (c2) Depth versus time. (c3) Magnitude versus time. The blue boxes in (a) and (c1) indicate the zoom-in for foreshock migration in Figure 5.

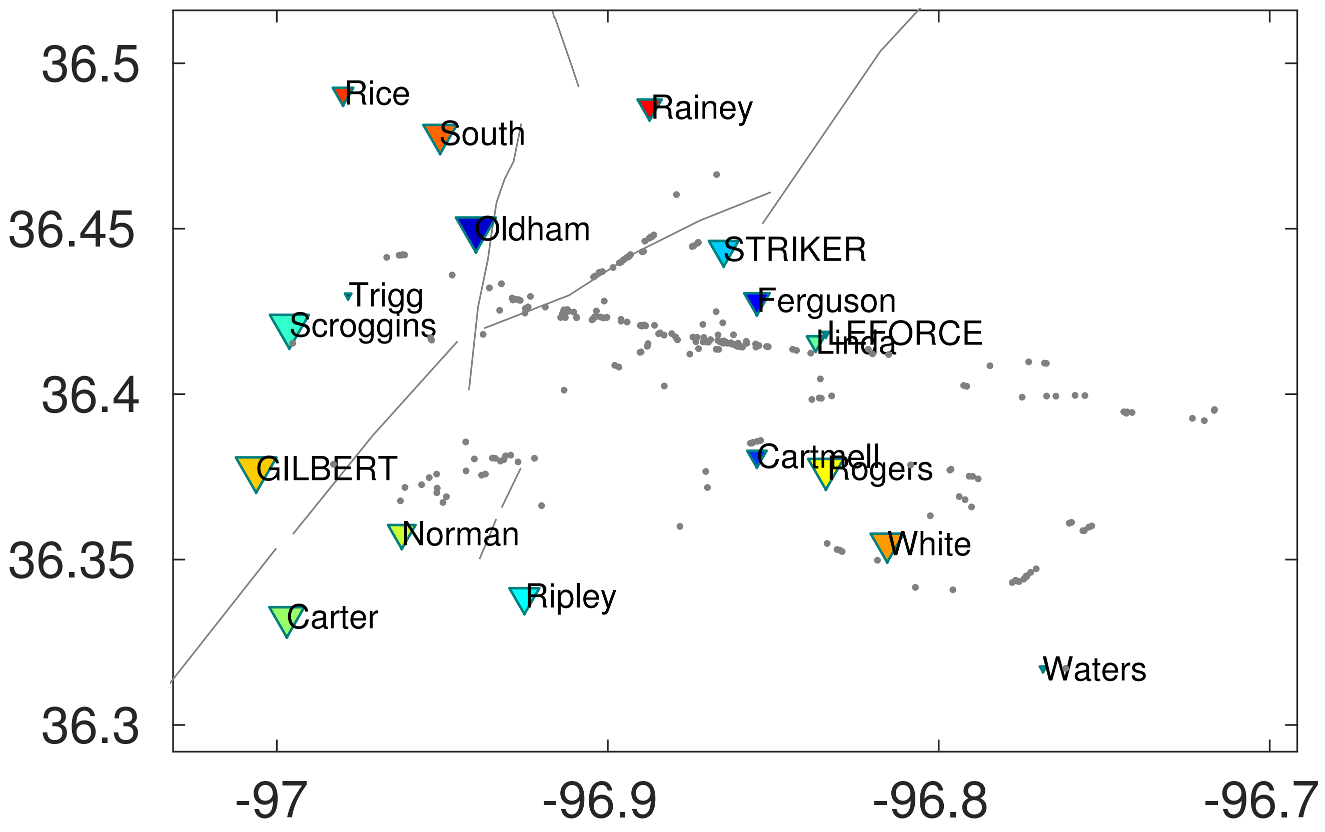

Figure S5. Map view of all the 18 wells included in the analysis shown in colored diamonds, with list of well names. The size of the triangles are corresponding to the average injection rate. Regional faults are shown in grey lines. Earthquakes are shown in grey dots. The data are obtained from the Oklahoma Corporation Commission.

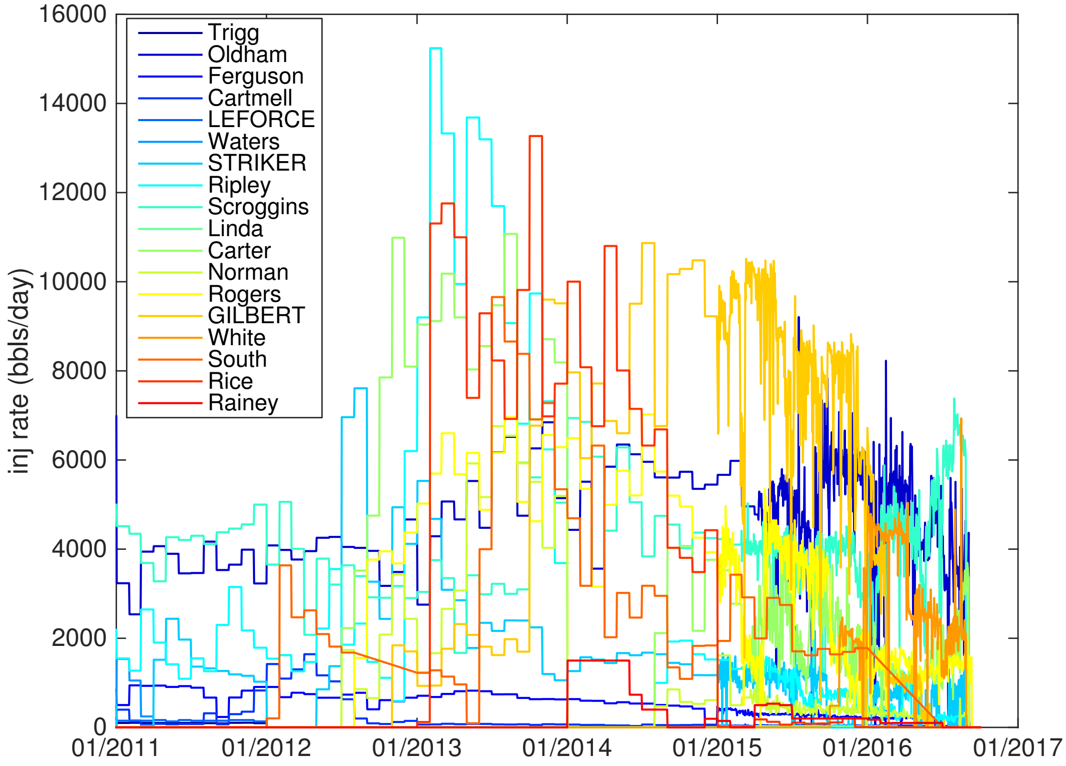

Figure S6. Injection histories for the daily injection rate of all 18 wells. Only monthly data are available before 2015, the monthly rate is divided by the number of days in a corresponding month to get daily rate. The colors correspond to the colors in Figure S5.

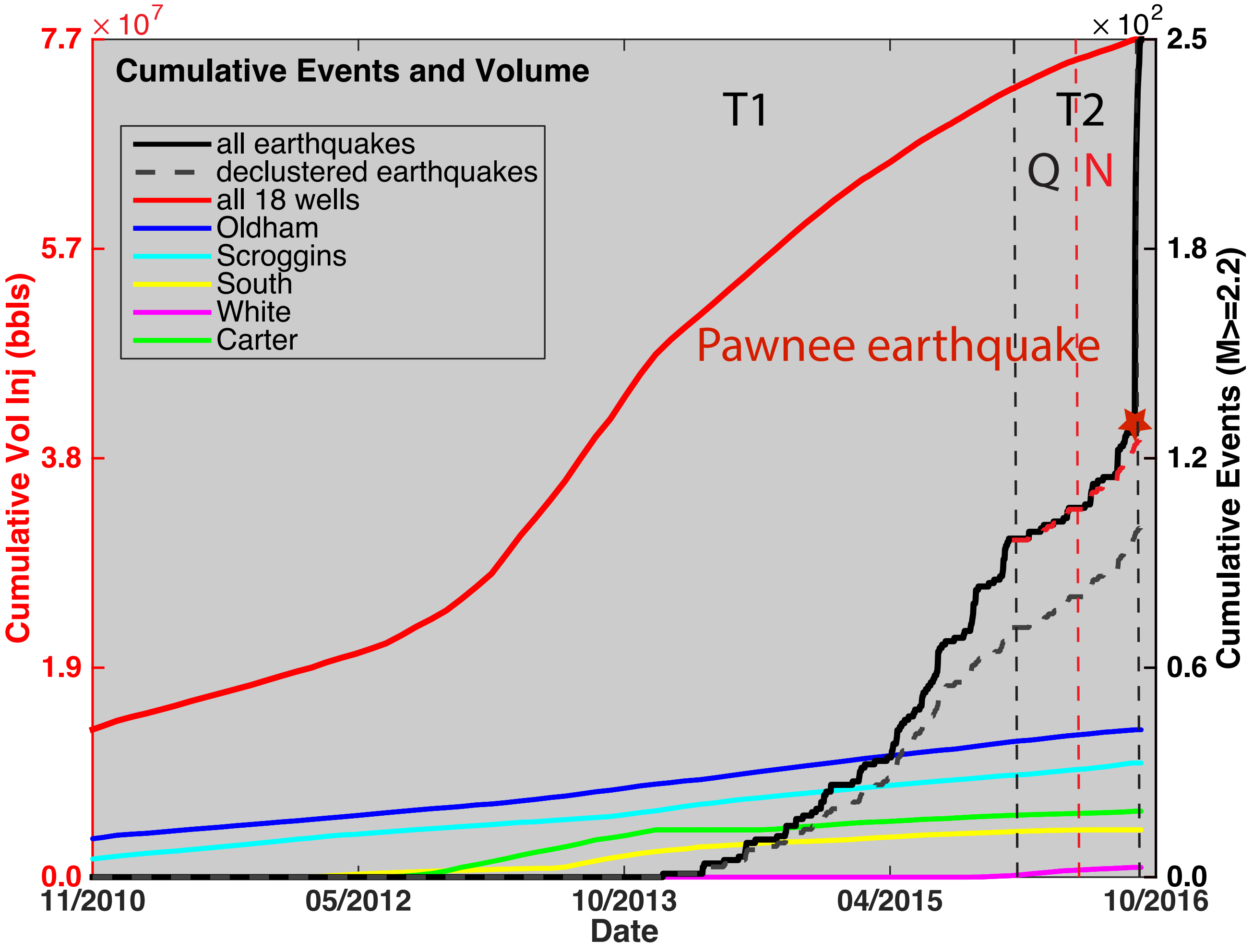

Figure S7. Cumulative number of injection volume for all wells combined (red) and selected individual wells. Cumulative number of earthquakes before declustering (black), and after declustering (grey –). The cumulative number from the declustered catalog is shifted to compare with the original catalog for T2 (red dashed line). The Pawnee earthquake is shown by red star. The names for individual wells are indicated in figure legend, and the colors are consistent with colors in Figures 4 and 6.

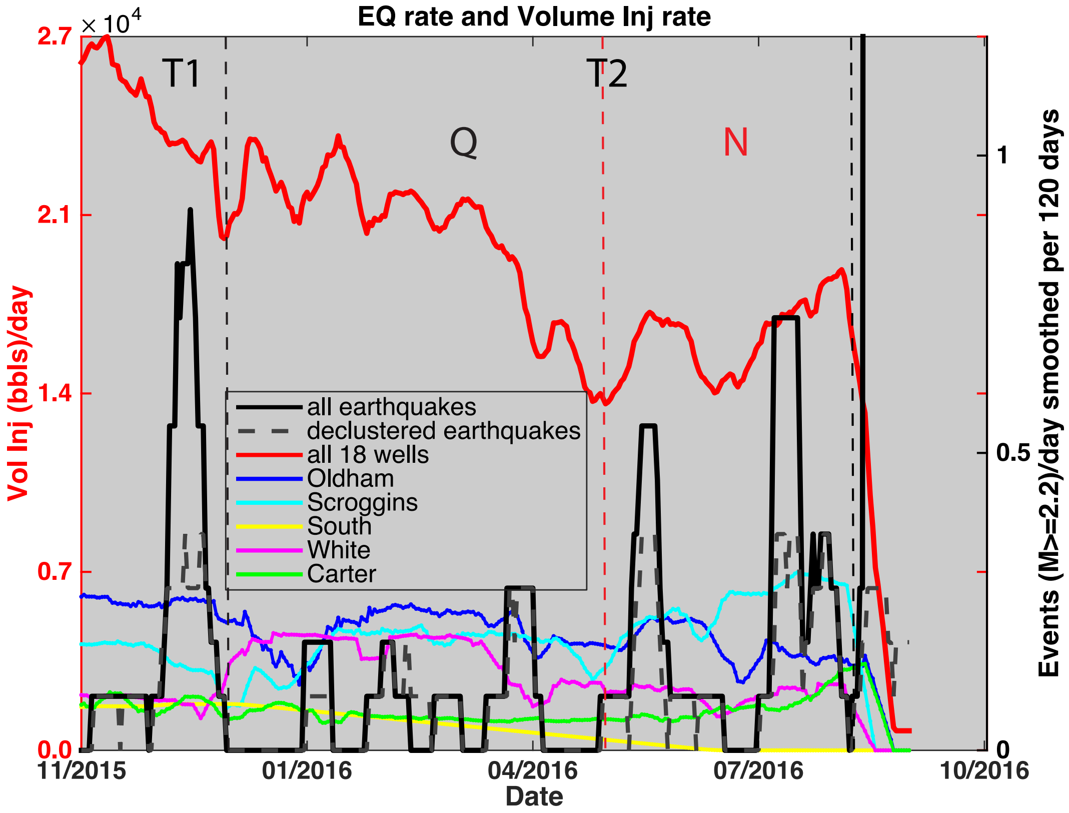

Figure S8. Similar to Figure 6, but for the time period between late 2015 and immediately before the mainshock, time period T2, using the same catalog as in Figure 6. This uses the relocated catalog.

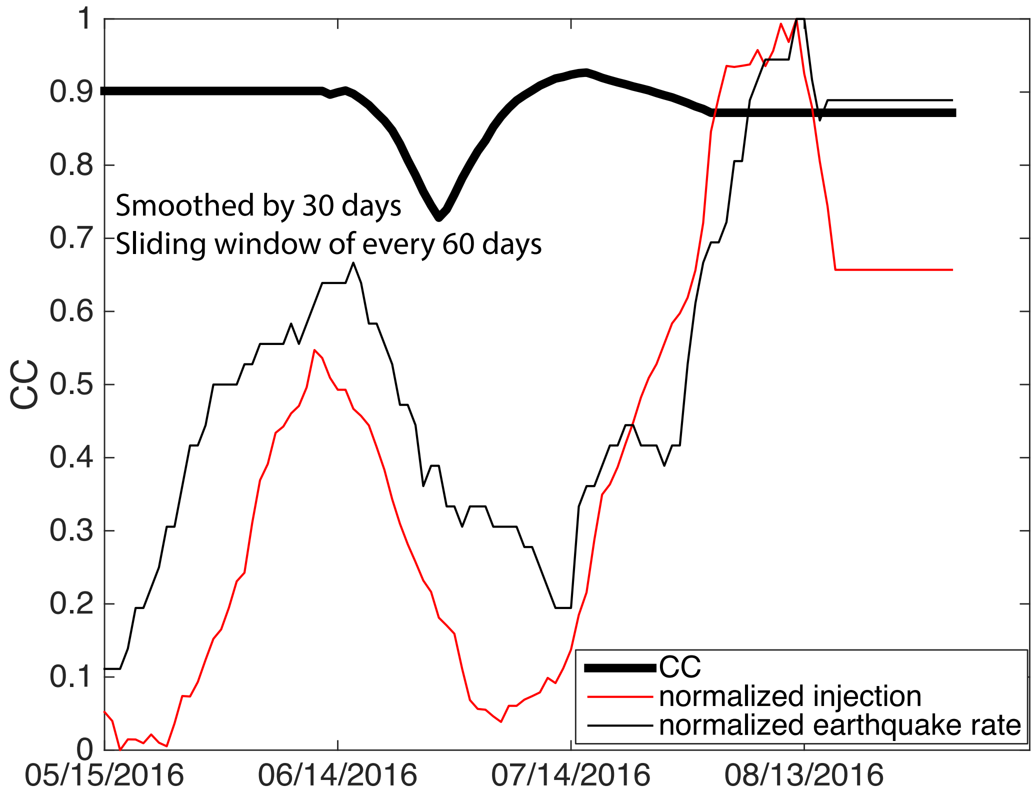

Figure S9. Similar to Figure 7, but with a 30-day smoothing window for data, and 60-day sliding window to calculate correlation variations. This uses the matched-filter catalog.

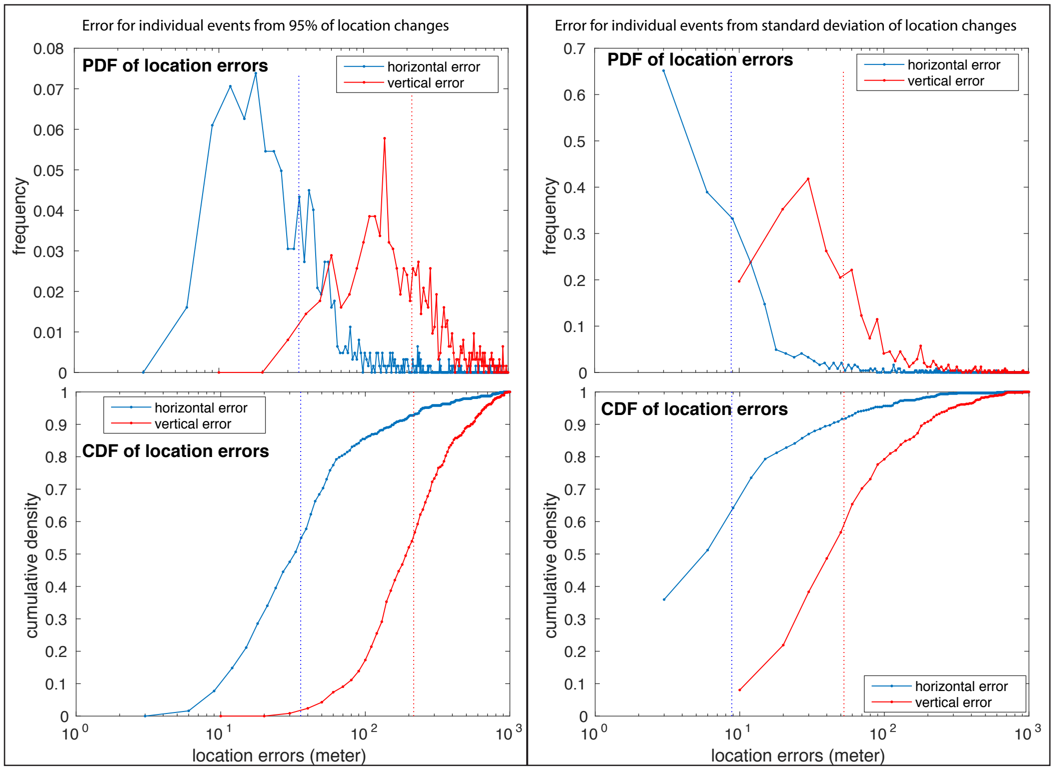

Figure S10. Probability density function and Cumulative density function of location error distributions. In all figures, blue is for horizontal error, red is for vertical error. The left column is taking the 95% of location changes from 50 bootstrap resampling as the location error for individual events. The right column is taking the standard deviation of location changes from 50 bootstrap resampling as the location error for individual events. In all figures, the dashed lines mark the median values for the location errors.

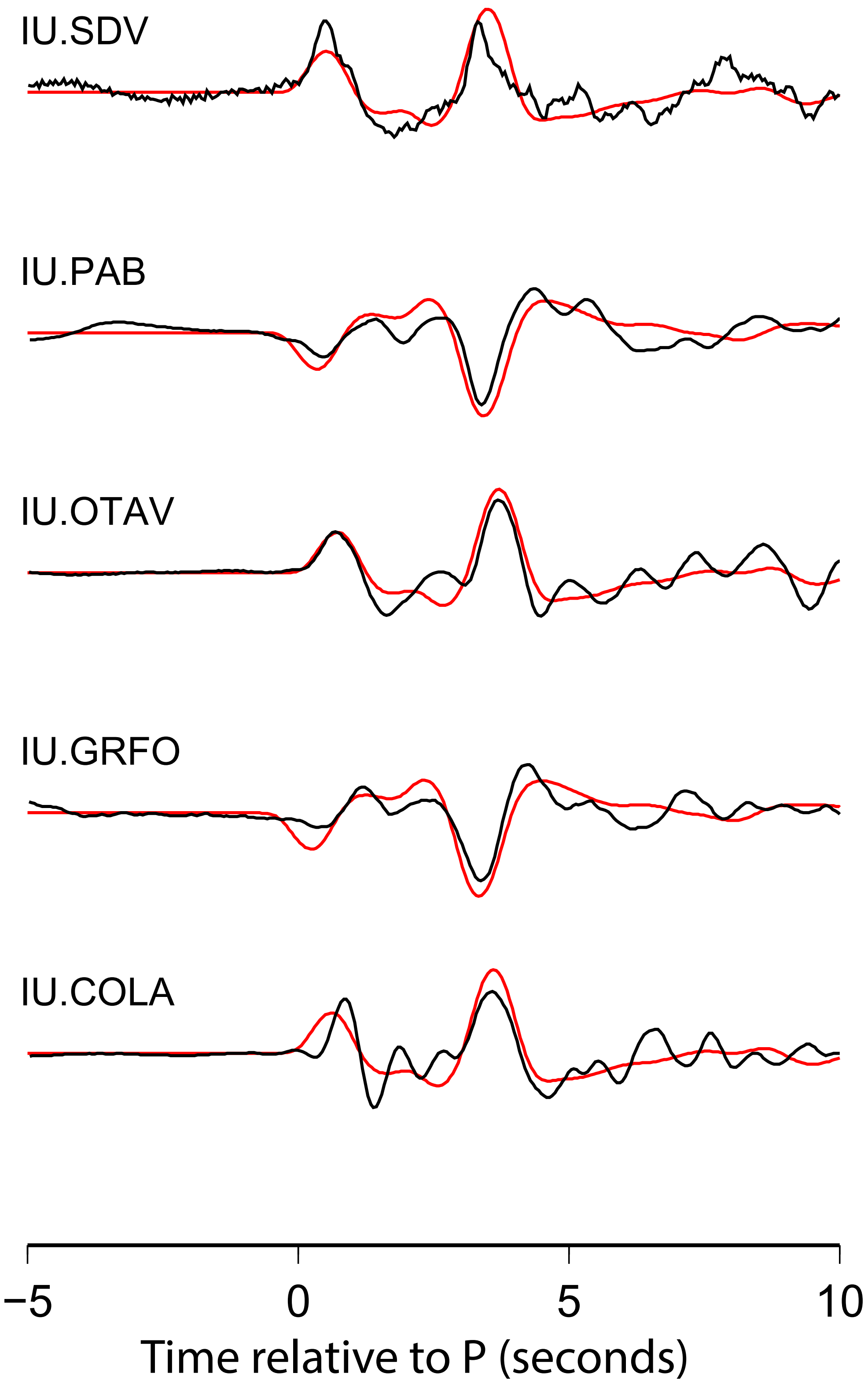

Figure S11. Waveform comparison for teleseismic stations. The black traces are observed broadband velocity seismograms, and red traces are synthetics calculated at centroid depth 7 km.
